# Knock-Down of the Phosphoserine Phosphatase Gene Effects Rather N- Than S-Metabolism in *Arabidopsis thaliana*

**DOI:** 10.3389/fpls.2018.01830

**Published:** 2018-12-11

**Authors:** Sladjana Samuilov, Nadine Rademacher, Dominik Brilhaus, Samantha Flachbart, Leila Arab, Stanislav Kopriva, Andreas P. M. Weber, Tabea Mettler-Altmann, Heinz Rennenberg

**Affiliations:** ^1^Chair of Tree Physiology, Institute of Forest Sciences, Faculty of Environment and Natural Resources, University of Freiburg, Freiburg, Germany; ^2^Institute of Plant Biochemistry, Cluster of Excellence on Plant Sciences, Heinrich Heine University, Düsseldorf, Germany; ^3^Botanical Institute, Cluster of Excellence on Plant Sciences, University of Cologne, Cologne, Germany; ^4^College of Science, King Saud University, Riyadh, Saudi Arabia

**Keywords:** amino acids, Cd treatment, cysteine, glutathione, glycine, phosphorylated pathway, serine

## Abstract

The aim of present study was to elucidate the significance of the phosphorylated pathway of Ser production for Cys biosynthesis in leaves at day and night and upon cadmium (Cd) exposure. For this purpose, *Arabidopsis* wildtype plants as control and its *psp* mutant knocked-down in phosphoserine phosphatase (*PSP*) were used to test if (i) photorespiratory Ser is the dominant precursor of Cys synthesis in autotrophic tissue in the light, (ii) the phosphorylated pathway of Ser production can take over Ser biosynthesis in leaves at night, and (iii) Cd exposure stimulates Cys and glutathione (GSH) biosynthesis and effects the crosstalk of S and N metabolism, irrespective of the Ser source. Glycine (Gly) and Ser contents were not affected by reduction of the *psp* transcript level confirming that the photorespiratory pathway is the main route of Ser synthesis. The reduction of the *PSP* transcript level in the mutant did not affect day/night regulation of sulfur fluxes while day/night fluctuation of sulfur metabolite amounts were no longer observed, presumably due to slower turnover of sulfur metabolites in the mutant. Enhanced contents of non-protein thiols in both genotypes and of GSH only in the *psp* mutant were observed upon Cd treatment. Mutation of the phosphorylated pathway of Ser biosynthesis caused an accumulation of alanine, aspartate, lysine and a decrease of branched-chain amino acids. Knock-down of the *PSP* gene induced additional defense mechanisms against Cd toxicity that differ from those of WT plants.

## Introduction

Cysteine (Cys) constitutes the initial product of sulfur assimilation that is used for protein biosynthesis and all anabolic pathways that require reduced sulfur. It is an important precursor of all thiol containing organic sulfur compounds such as glutathione (GSH) (Noctor et al., 2012), methionine (Met) (Hesse et al., 2004; Wirtz and Droux, 2005), and glucosinolates (GLS) (Falk et al., 2007). Many studies showed the importance of Cys and GSH in the defense of plants against oxidative stress (Noctor et al., 2012), including biotic stress by bacterial, fungal, and herbivore attacks (Hernández et al., 2017) and abiotic stress such as high temperature (Almeselmani et al., 2006), drought, and heat (Rennenberg et al., 2006) as well as heavy metal exposure (Lima et al., 2006; Gill et al., 2012). With its thiol group, GSH also plays an important role in scavenging reactive oxygen species (ROS) in the ascorbate-glutathione cycle (Noctor et al., 2012), in the detoxification of xenobiotics by conjugation *via* GSH-S transferases (Gullner et al., 2001), and for the sequestration of heavy metals as metabolic precursor of phytochelatins that function as heavy metal chelators (Herbette et al., 2006; Lima et al., 2006). Besides GSH, Met is another important sink of Cys that is used for the synthesis of proteins, S-adenosyl methionine (SAM), and S-methylmethionine (SMM) (Bourgis et al., 1999). Further, glucosinolates are secondary sulfur-rich, cys-containing metabolites characteristic for *Brassicaceae* (Huseby et al., 2013) that protect plants against herbivores and pathogens (Halkier and Gershenzon, 2006).

The sulfur in Cys originates from sulfate that is mostly reduced in the chloroplasts (Takahashi et al., 2011). Prior to its reduction, sulfate is activated by adenylation to adenosine 5′-phosphosulfate (APS) in a reaction catalyzed by ATP sulfurylase (ATPS; EC 2.7.7.4) (Takahashi et al., 2011). After activation, APS is reduced to sulfide in a two-step procedure (Takahashi et al., 2011). Part of the sulfide produced exclusively in chloroplasts/plastids can be transported to other cellular compartments of Cys synthesis, i.e., the mitochondria and the cytosol, possibly by diffusion of H_2_S through the chloroplast and mitochondria membranes (Jacques, 1936). Beside sulfide, serine (Ser) is the second precursor of Cys synthesis. In the first step, Ser together with acetyl-CoA is used for the synthesis of O-acetylserine (OAS) by serine acetyltransferase (SAT). In the second step, *O*-acetyl-serine (thiol) lyase (OAS-TL) replaces the activated acetyl moiety in OAS by sulfide and releases Cys (Takahashi et al., 2011; Rennenberg and Herschbach, 2014). Thus, Ser provides the carbon and nitrogen backbone required for Cys synthesis (Kopriva and Rennenberg, 2004). Therefore, Cys synthesis requires a cross-talk between sulfur, carbon and nitrogen metabolism (Kopriva and Rennenberg, 2004).

The nitrogen in Ser is a product of nitrogen reduction and assimilation, while the carbon skeleton originates from 3-phosphoglycerate (3-PGA), an intermediate of glycolysis and/or from 2-phosphoglycolate, a product of photorespiration (Kopriva and Rennenberg, 2004). Since nitrogen assimilation is catalyzed by the GS-GOGAT pathway that requires 2-oxoglutarate for glutamate synthesis, reactions of the tricarboxylic acid (TCA) cycle in the mitochondria are also involved in providing Ser for Cys synthesis (Kopriva and Rennenberg, 2004). Ser biosynthesis takes place in mitochondria, chloroplasts/plastids, and the cytosol by three distinct alternative routes, one photorespiratory and two non-photorespiratory pathways. The latter include the phosphorylated and the glycerate pathway of Ser synthesis (Ros et al., 2014). Ser production in mitochondria, catalyzed by the reactions of the glycine decarboxylase multi-enzyme complex (GDC) and hydroxymethyltransferase (SHMT) in the photorespiratory pathway, is currently considered to constitute the most important biosynthetic route of Ser synthesis in photosynthetic cells (Voll et al., 2006; Engel et al., 2007, 2011; Eisenhut et al., 2013). The *Arabidopsis* A BOUT DE SOUFFLE (*bou*-*2*) knockout mutant of the mitochondrial BOU protein showed reduced GDC activity and accumulation of Gly and Ser upon growth in ambient air (Eisenhut et al., 2013; Samuilov et al., submitted). Although *bou*-*2* mutants are not lethal, their growth is arrested at ambient air, but restored after 2 days of growth at elevated CO_2_ (Eisenhut et al., 2013). Apparently, suppression of photorespiration by elevated CO_2_ is required for normal growth of the mutant (Eisenhut et al., 2013). However, in the *Arabidopsis PsP-L* mutant, overexpressing the L-protein (mtLPD; EC 1.8.1.4.) of the GDC enzyme complex, the Gly content, but not the Ser content was reduced compared to wild type plants, indicating accelerated turnover of Gly (Timm et al., 2015). In addition, the Cys and GSH content significantly increased in the PsL-L2 line of *PsP-L* mutant (Timm et al., 2015).

The phosphorylated pathway has been proposed to constitute an important route of Ser biosynthesis during embryo development, in non-photosynthetic tissue such as roots, in photosynthetic tissue during the night (Ros et al., 2014 and their references), and under conditions of suppressed photorespiration (Benstein et al., 2013). Five days of high CO_2_ exposure of Arabidopsis mutants with a silenced phosphoglycerate dehydrogenase gene (*PGDH1*) led to strong inhibition of growth and a decrease in the Ser content, while the *PGDH1* gene in control plants exhibited enhanced expression when grown at elevated CO_2_ (Benstein et al., 2013). Further, 8 h darkness induced the expression of the *PGDH1* and *PGDH3* genes in photosynthetic tissue of *Arabidopsis* plants, while in roots the expression of all three *PGDH* isoforms was comparable at day and night (Toujani et al., 2013). These results support the view that the phosphorylated pathway is an important source of Ser in autotrophic tissues in the dark and in heterotrophic tissues. In addition, the phosphorylated pathway has been proposed to play an important role in plants under environmental stresses (Ho and Saito, 2001). The expression of genes of phosphorylated pathway such as *PGDH1, PGDH2*, and *PSAT1* was induced in plants at the site of infection with the necrotrophic fungal pathogen *Botrytis cinerea* (Benstein et al., 2013), where enhanced Cys synthesis is required for the hypersensitive response to this pathogen (Foyer and Rennenberg, 2000). Suitable mutants for studying the significance of the plastidial phosphorylated pathway of Ser synthesis and Cys production are *Arabidopsis PSP* gene knock-down mutants of the phosphoserine phosphatase gene (*PSP*; At1g18640). Compared to the *Arabidopsis PSP* gene loss of function mutant (Benstein et al., 2013), the mutant with the repressed expression of the *PSP* gene is not embryo lethal.

The aim of the present study was to elucidate (1) the significance of chloroplastic Ser production for Cys biosynthesis in mature leaves during daylight and darkness and its use for protein, GSH and GLS synthesis, (2) the role of plastidal versus photorespiratory Ser supply for Cys synthesis at enhanced Cys demand upon Cd exposure, and (3) the effect of Cd on the sulfur (S) and nitrogen (N) cross-talk for Cys synthesis. Experiments were conducted with *Arabidopsis* wild type (WT) plants and newly generated *PSP* gene knock-down mutants of *PSP*. It was hypothesized that photorespiratory Ser is the dominant precursor of Cys synthesis in autotrophic tissue in the light (hypothesis 1), but the chloroplastic Ser production takes over in autotrophic tissue at night (hypothesis 2). In addition, it was assumed that Cd stimulates Cys and GSH biosynthesis and interacts with the cross-talk of S and N metabolism, irrespective of the source of Ser (hypothesis 3).

## Materials and Methods

### Generation and Selection of the Arabidopsis *PSP* Knock-Down Mutant

The RNAi construct for the generation of PSP knock-down plants was designed according to Guo et al. (2003) using the intron-containing vector pSK-int as intermediate vector for cloning. The 660 bp long DNA fragments encoding for the sense and antisense RNA of the *Arabidopsis thaliana PSP* gene (At1g18640, nt 3-663 on cDNA) were generated using primer pairs P1/P2 and P3/P4, respectively, and were integrated into the pSK-Int vector via internal restriction sites (Supplementary Table S1). The complete construct was cloned into the final plant vector pUT-Kan encoding an Ubiquitin 10 promoter for expression of the construct and a kanamycin resistance gene for plant selection (Supplementary Figure S1A). Transformation in *Arabidopsis thaliana* Col-0 wildtype plants was carried out using the floral dip method (Clough and Bent, 1998). Transformed plants were selected on half-strength Murashige-and-Skoog medium plates containing 50 μg/ml kanamycin. Correctness of insert was verified via PCR on genomic DNA using primer P5 and P6. Selection was done out of 8 independent lines and was based on reduction of *PSP* transcript abundance. Knockdown of *PSP* mRNA amount was measured by RT-PCR on cDNA of T1 plants using primer P7 and P8. RT-PCR of *psp* mutant lines revealed a strong reduction of the *PSP* transcript abundance in mutant line #17, while mutant line #18 showed a weaker reduction in *PSP* transcript abundance. Detailed results of *PSP* knockdown amount for mutant line #17 and #18 in comparison to the WT at day and night generated via transcriptome analysis are shown in Supplementary Figure S1B. Homozygous T3 plants of *psp* mutant lines #17 and #18 were used for further characterization of the mutants.

### Plant Material and Growth Conditions

For the characterization of the mutant, chlorine gas surface-sterilized seeds of *Arabidopsis thaliana* Col-0 wildtype and *psp* mutant lines #17 and #18 were stratified at 4°C for 2 days. Germination was carried out on half-strength Murashige-and-Skoog (MS) medium (Duchefa Biochemie) containing 0.8% agar (w/v) in growth chambers (12 h light/12 h dark, 22°C day temperature/18°C night temperature, 100 μmol photons m^-2^ s^-1^ light intensity, ambient air). After pre-incubation of *psp* mutant plants and Col-0 wildtype control plants on ½ MS plates for 14 days, plants were transferred to soil. Further plant growth occurred in growth chambers under long day conditions (16 h light/8 h dark, 22°C day temperature/18°C night temperature, 100 μmol photons m^-2^ s^-1^ light intensity) in high CO_2_ (3000 ppm CO_2_). The growing condition allowed us to compare results of *psp* mutants with the *bou-2* mutant. 24 h before harvest, plants were shifted to ambient air conditions. Other growth parameters remained unchanged.

To examine the significance of the phosphorylated pathway of Ser biosynthesis for Cys synthesis under normal conditions and upon Cd treatment, *Arabidopsis thaliana* Col–0 and *psp* mutant line #17 were used. Prior to cultivation, seeds were surface sterilized with 70% (v/v) ethanol containing 1% (v/v) Triton X-100, twice with 100% ethanol, sown on soil, and incubated at 4°C for at least 2 days to break dormancy. After incubation, seeds were transferred to growth chambers and kept under controlled conditions (12 h light/12 h dark, 22°C day temperature/19°C night temperature, 100–150 μmol photons m^-2^ s^-1^, light intensity, 60% relative humidity), at ambient CO_2_. After emergence of two fully developed leaves, each plant was transferred into a separate pot with new soil substrate and cultivated 4 weeks under the conditions indicated above. One week before harvest, one set of plants was watered with 50 mL 2mM CdCl_2_ solution in one dose. Experiments were performed with 5-week old plants.

### Experimental Design

For the characterization of *psp-17* and *psp-18* mutant transcriptome and metabolite analysis were performed using whole rosettes of 4 plants per mutant line and wildtype harvested after 4 weeks of cultivation. Day samples were taken 10 min after light onset, night samples 3 h before light onset and were snap-frozen in liquid nitrogen.

Different sets of plants were harvested for [^35^S] sulfate feeding experiments and metabolite analyses. For [^35^S] sulfate feeding experiments, both Cd-treated and non-treated plants were harvested at two time points, i.e., 3 h after light onset and 5 h before light onset. Fully mature leaves (leaf number 5 to 7 determined by the order of emergence from the rosette) were cut and half of the leaves were used for analysis of flux of ^35^S into the pools of sulfate, proteins, GSH and GLS, as well as GSH and GLS contents. The other half of the leaves was treated in the same way with non-radioactive Hoagland solution and used for the determination of sulfate, proteins and total thiol contents. At the end of [^35^S] sulfate feeding experiments, leaves were frozen in liquid nitrogen and stored at -80°C until further analysis. For metabolite analyses, additional sets of Cd treated and non-treated plants were harvested at day (3 h after light onset) and night (5 h before light onset). The whole rosette of the plants was used for quantification of metabolites and frozen immediately after harvest in liquid nitrogen. Plant material was stored at -80°C until analyses.

### RNA Extraction, Preparation, and Sequencing of Illumina Libraries

All RNA-Seq experiments were carried out on three independent biological replicates. Whole rosettes were harvested and immediately frozen in liquid nitrogen. RNA was isolated from ground tissue using the RNeasy Plant Mini Kit (QIAGEN, Hilden, Germany). Residues of DNA were removed with DNase (New England Biolabs, Ipswitch, MA, United States). RNA integrity, sequencing library, and fragment size were analyzed on a 2100 Bioanalyzer (Agilent Technologies, Santa Clara, CA, United States). Libraries were prepared using the TruSeq RNA Sample Prep Kit v2 (Illumina, San Diego, CA, United States) and quantified with a Qubit 2.0 (Invitrogen, Waltham, MA, United States). Samples were multiplexed with 12 libraries per lane and sequenced in paired-end mode (Rapid Run, 150 bp read length) on an Illumina HiSeq 3000 platform, yielding on average ∼26 million reads per library.

### Read Mapping and mRNA-Seq Data Analysis

After successful quality control with the Fast QC software^1^ (v0.11.5), Illumina reads were quantified by mapping against the Arabidopsis TAIR10 reference transcriptome including primary and secondary transcripts^2^ using Kallisto v 0.43.0 (Bray et al., 2016) in default mode with 30 times bootstrapping for sleuth. Kallisto provides normalized gene expression in transcripts per million (tpm). Primary transcripts for graphical presentation were chosen based on highest average tpm in the WT.

### Metabolite Measurements

Aliquots of 50–80 mg frozen leaf material were extracted with a mixture of chloroform-methanol-water for analysis of sugars, organic acids and amino acids by gas chromatography-mass-spectrometry (GC-MS) according to Fiehn et al. (2000) using a 7200 GC-QTOF (Agilent, Milford, CT, United States). Peak integration was conducted with MassHunter Software from Agilent. For relative quantification, metabolite peak areas were normalized to the amount of extracted plant material and the peak area of the internal standard ribitol added to the extraction solution.

### [^35^S] Sulfate Feeding Experiments

For [^35^S] sulfate feeding experiment, leaves were transferred into tubes (REF. 60. 732, Sarstedt AG & Co., Nümbrecht, Germany) with 10 ml Hoagland solution (5 mM KNO_3_, 5 mM Ca(NO_3_)_2_, 2.5 mM KH_2_PO_4_, 2.5 mM KOH, 25 μM MnCl_2_, 2.5 μM ZnSO_4_, 1.25 μM CuSO_4_, 0.075 mM H_3_BO_3_, 2.5 μM Na_2_MoO_4_, 1.25 μM Co(NO_3_)_2_, 0.0675 mM Fe-EDTA) adjusted to 0.2 mM SO_4_^2-^ for 2 h of pre-incubation. Thereafter, leaves were placed in a fresh Hoagland solution containing radioactively labeled [^35^S]-SO_4_^2-^ (S-RA-1, Hartman Analytic GmbH, Braunschweig, Germany) at 50 μCi (1.85 × 10^6^Bq) for day and 100 μCi (3.7 × 10^7^Bq) for night exposures. At the beginning of the 4 h incubation, leaves were placed in a desiccator and infiltrated with a vacuum pump to exchange air from the apoplastic space with radioactive Hoagland solution. At the end of exposure, sulfate uptake was stopped by washing leaves with 10 ml incubation solution. For night experiments, pre-incubation and incubation were performed in the dark; night harvests were carried out under green light (GreenHornet LED, 3.5W, 480 Lumen; Dutch-Headshop Services, Beverwijk, Netherlands). Leaf tissue was homogenized with a micro pestle and mortar and extracted with 0.1 M HCl at a ratio of 1:10 (w/v). An aliquot of 10 μl extract was mixed with 2 ml of Rotiszint eco plus scintillation cocktail (Carl Roth GmbH + Co. KG, Karlsruhe, Germany) and radioactivity was determined with a scintillation counter (LS 6500 Multi-Purpose Scintillation Counter, Beckman Coulter, Brea, CA, United States). From the data obtained and the specific radioactivity of the incubation solution, total sulfate uptake was calculated.

### [^35^S]-Metabolite Analyses and Determination of the Flux Through the Sulfate Assimilation Pathway

#### Protein Content and [^35^S] Flux Into Protein

The preparation of samples for protein determination was done as described by Samuilov et al. (2018). The protein content was determined as described by Bradford (1976) using 1 mg ml^-1^ bovine serum album (BSA) as a standard (ranging from 0.2 to 1 mg ml^-1^). To determine radioactivity in proteins, aliquots of 50 μl of the supernatants previously extracted with 0.1 M HCl were used. Protein was precipitated with 12.5 μl 100% trichloroacetic acid (TCA) for 15 min at ice, washed with 200 μl 100% ethanol and dissolved in 100 μl 0.1 M NaOH as described by Kopriva et al. (1999). Radioactivity was determined in 200 μl supernatant after mixing with 2 ml Rotiszint eco plus scintillation cocktail (Carl Roth GmbH + Co. KG, Karlsruhe, Germany).

#### Thiol Composition and Content and [^35^S] Flux Into Thiols

The content of thiols was determined using aliquots of 25 μl supernatant previously extracted with 0.1 M HCl and neutralized by adding an equal volume of 0.1 M NaOH. The extraction and quantification of thiols followed the protocol described by Schupp and Rennenberg (1988) and modified by Strohm et al. (1995). Thiols were derivatized with monobromobimane (5 μl, 100 mM) and stabilized by adding 100 μl acetic acid (9%, v/v) after 15 min of derivatization in the dark at 37°C. Aliquots of 100 μl derivatized solution were injected into an HPLC system (Dionex UltiMate 3000; Thermo Fisher Scientific GmbH; Waltham, MA, United States) and thiol derivatives were separated on a Spherisorb ODS2 (C_18_; 250 × 4.6 mm, 5 μm particle size) column (Waters Corporation, Milford, CT, United States) using a solution of 0.25% (v/v) acetic acid and 10% (v/v) methanol as buffer A and 0.25% acetic acid and 90% (v/v) methanol as buffer B. Thiols were detected fluorometrically (Waters 474 Fluorescence detector, Waters Corporation, Milford, CT, United States) with excitation at 390 nm and emission at 480 nm. The [^35^S]SO_4_^2-^ incorporation into thiols was determined by a radioactivity detector module (Radio Flow Detector FlowStar LB 513; Berthold Technologies GmbH & Co. KG, Bad Wildbad, Germany) connected to the HPLC.

#### Glucosinolate Analysis and [^35^S] Flux Into Glucosinolates

The [^35^S]SO_4_^2-^ incorporation into glucosinolates (GLS) and total GLS content was determined using ∼50 mg of frozen plant material. The extraction and quantification of GLS followed the protocol described by Brown et al. (2003). Quantification was based on UV absorption at 229 nm and response factors relating to the internal standard. GLS were determined by HPLC (Dionex UltiMate 3000; Thermo Fisher Scientific GmbH; Dreieich, Germany) by separation on a Spherisorb ODS2 (C18; 250 × 4.6 mm, 5 μm particle size) column, using distilled water (solvent A) and 100 % acetonitrile (solvent B) for elution. To determine [^35^S]SO_4_^2-^ incorporation into GLS, 300 μl supernatant were mixed with 3 ml Rotiszinteco plus scintillation cocktail and radioactivity was determined by scintillation counting.

#### Quantification of Sulfate

Sulfate was extracted and determined from ∼30 mg leaf tissue (homogenized under liquid nitrogen) by anion exchange chromatography as described by Herschbach et al. (2000). For analysis an automatic ion analyzer (DX 120, Dionex Corporation, Sunnyvale, CA, United States), equipped with an IonPac^TM^ column (AS9-SC, 4 × 250 mm; Dionex, Thermo Fisher Scientific GmbH; Waltham, MA, United States) was used. Anions were eluted with a mixture of 2.0 mM Na_2_CO_3_ and 0.75 mM NaHCO_3_. Sulfate was detected by a conductivity detector module (CDM, Dionex Corporation, CA, United States).

#### Quantification of Total Non-Protein Thiols

For the determination of total non-protein thiols (NPT), a modified method of Queval and Noctor (2007) was applied. Total thiols in leaf extract were assayed as 5,5′-dithio-bis-[2-nitrobenzoic acid]-reactive thiols (DTNB-reactive thiols) by spectrophotometry (Beckman UV-DU650, Beckman Coulter, United States) using glutathione (GSH) as a standard. Approximately 100 mg frozen leaf powder was extracted in 1 ml 0.2N HCl. Aliquots of 0.5 ml supernatant were transferred into fresh micro tubes (Sarstedt AG & Co., Nümbrecht, Germany) and neutralized with 0.4 ml 0.2 M NaOH in the presence of 50 μl 0.2 M NaH_2_PO_4_ (pH 5.6). For thiol quantification by spectrophotometry, each semi-micro cuvette (Sarstedt AG & Co., Nümbrecht, Germany) contained 500 μl phosphate-EDTA buffer (0.2 M NaH_2_PO_4_, pH 7.5; 10 mM EDTA), 50 μl of 12 mM DTNB and 450 μl neutralized sample extract (total volume 1 ml). For standards, the extract was replaced by 450 ml of 0, 10, 20, 30, 40, 50 μmol GSH. The absorbance was measured at a wavelength of 412 nm 3 min after addition of extract or standard.

### Statistical Analysis

#### Statistical Analyses of Metabolites and mRNA-Seq Data for Characterization of *psp* Mutants

Student’s *t*-test was performed to determine significant differences between metabolite amount of WT and the mutants. Wald test implemented in sleuth v0.28.1 (Pimentel et al., 2016) was employed to test for differential gene expression between WT and mutants at day and night. If not indicated otherwise, *p*-values were corrected for multiple sampling by Benjamini–Hochberg correction (Benjamini and Hochberg, 1995) as implemented in sleuth and an alpha of 0.01 was chosen. Log2 expression ratios between genotypes or exposures were calculated after addition of a pseudo-count of 1 to prevent infinite values.

Principle component analysis (PCA, Supplementary Figure S2) performed independently on both metabolite and mRNA-Seq data revealed an obvious outlier in both datasets (i.e., one biological replicate of *psp*-18 at night, data not shown). This outlier was removed. PCA was done with function prcomp() implemented in the R statistics software. The graphic work was performed in R statistics software (R version 3.3.0 provided by the CRAN project^3^).

#### Statistical Analyses of Metabolite and ^35^[S] Flux Data

To statistically analyze metabolite and ^35^[S] flux data of *Arabidopsis* Col-0 and its *psp-17* mutant the software package SigmaPlot V. 11.0 (Systat Software GmbH, Erkrath, Germany) was used. To determine significant differences between the WT and the *psp* mutant at the same time point and treatment, Student’s *t*-test was performed. To determine differences between time points and differences between control and treatment of the same plant type, One-way Anova was conducted in combination with Tukey correction. If normality or equal variance failed, Student’s *t*-test or One-way Anova on Ranks was performed. All data are presented as mean value ± standard deviation of 3 independent replicates. The graphic work was performed in OriginPro V.9.1. (Additive GmbH, Friedrichsdorf, Germany). Venn diagrams were used to present metabolite abundances shared between genotypes, time of exposure and treatments^4^.

### Accession Numbers

The read data have been submitted to the National Center for Biotechnology Information Gene Expression Omnibus under accession number GSE112254^5^.

## Results

### Characterization of *Arabidopsis PSP* Gene Knock-Down Mutants

Principal component analysis (PCA) showed a clear separation between metabolite amounts of WT plants and the two mutants at day and night, as well as transcript amounts at day and night, except for a slight overlap between WT and *psp-18* line at transcript level at day (Supplementary Figure S2). Apparently, both mutants display similar differences to the WT. The slight overlap of transcript amounts between the WT and the *psp-18* line, but not with the *psp-17* line, is in agreement with weaker reduction of *PSP* transcript abundance in the *psp-18* compared with the *psp-17* line (Supplementary Figure S1B). In addition, the mild effects observed in *psp-18* mutant might be due to a low decrease in *psp* enzyme activity in comparison to WT.

**FIGURE 1:**
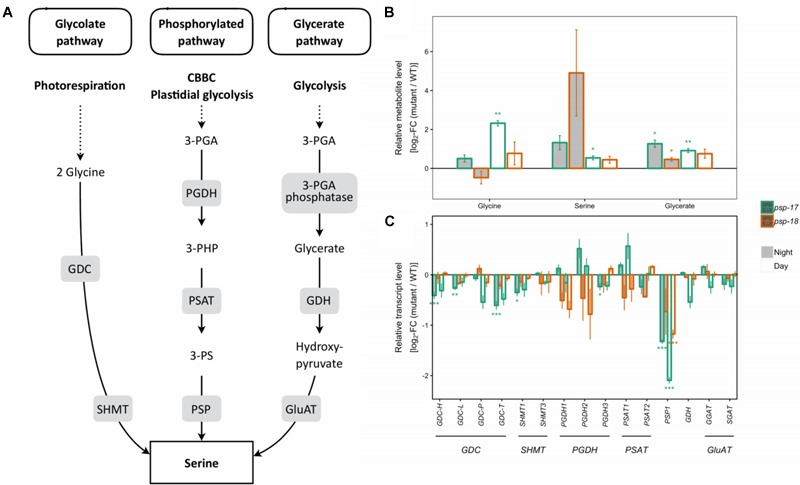
Photorespiratory and non-photorespiratory pathways of serine biosynthesis. **(A)** Schematic presentation of three pathways of serine biosynthesis (adapted from Ros et al., 2013). **(B)** Metabolite amounts in *psp* mutants (*n* = 4, except *psp-18* night: *n* = 3) relative to the wt. Asterisks indicate significant differences between respective mutant and wt as determined by Student’s *t*-test (^∗^*p* < 0.05, ^∗∗^*p* < 0.01, and ^∗∗∗^*p* < 0.00). **(C)** Transcript abundances in *psp* mutants (*n* = 3, except *psp-18* night: *n* = 2) relative to the wt. Asterisks indicate differential abundance between respective mutant and wt as determined by Sleuth (^∗^FDR < 0.05, ^∗∗^FDR < 0.01, and ^∗∗∗^FDR < 0.001). GDC, Glycine decarboxylase; SHMT, Serine hydroxymethyltransferase; PGDH, Phosphoglycerate dehydrogenase; PSAT, 3-phosphoserine aminotransferase; PSP, 3-phosphoserine phosphatase; GDH, Glycerate dehydrogenase; GluAT, Hydroxypyruvate aminotransferase.

In both mutant lines, transcripts encoding all isoforms of GDC and SHMT genes of the photorespiratory pathway were lower compared to the WT at day and night, with significant differences observed for the *psp-17* mutant line (Figure 1). In addition, transcript abundance of genes encoding for one or multiple isoforms of seven key enzymes and transporters of photorespiration were reduced in both mutant lines relative to WT, again with significant differences observed for *psp-17* line (Supplementary Figure S3). Genes of the phosphorylated pathway of Ser biosynthesis did not show any significant changes in transcript abundance in the mutants except for a significant downregulation of the *PGHD3* isoform at night and *PSP1* at day and night in the *psp-17* line. Although the decrease in transcript amount was observed for both mutant lines relative to WT, the steady state pool size of photorespiratory metabolites (Gly, Ser, glycerate) was significantly higher in the *psp-17* mutant line relative to the WT at both day and night (Figure 1). Although not significant, the same trend was observed for the *psp-18* line except for Gly at night.

**FIGURE 2:**
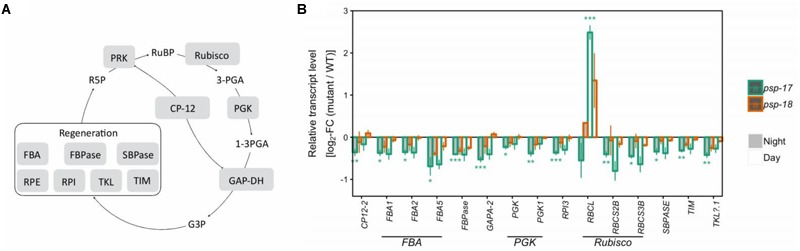
The Calvin-Benson-Bassham-cycle (CBBC). **(A)** Schematic presentation. **(B)** Transcript abundances in *psp* mutants (*n* = 3, except *psp-18* night: *n* = 2) relative to the wt. Asterisks indicate differential abundance between respective mutant and wt as determined by Sleuth (^∗^FDR < 0.05, ^∗∗^FDR < 0.01, and ^∗∗∗^FDR < 0.001). Only significantly different data between either *psp* mutant and wt at either time point is shown. Additional transcripts are presented in Supplementary Dataset S1. CP12, CP12 domain-containing protein; FBPase, fructose-bisphosphatase; GAPDH, glyceraldehyde 3-phosphate dehydrogenase; PGK, phosphoglycerate kinase; PRK, phosphoribulokinase; RPE, ribulose-phosphate 3- epimerase; RPI, ribose-5-phosphate isomerase; Rubisco, ribulose-bisphosphate carboxylase; SBPASE, sedoheptulose-1,7-bisphosphatase; TKL, transketolase.

Genes of the CBB cycle were significantly downregulated at both day and night in *psp-17* except for the plastid-encoded Rubisco subunit *RBCL* that showed significant 2.5-fold higher transcript abundance at day relative to the WT (Figure 2). Although not significant, the same trend was observed in the *psp-18* line. Sugar contents did not show any significant changes in both mutant lines relative to the WT except for significant increase in sucrose amount at both day and night in the *psp-17* line (Supplementary Dataset S2). Although not significant, similar increase in sucrose was observed for the *psp-18* line.

**FIGURE 3:**
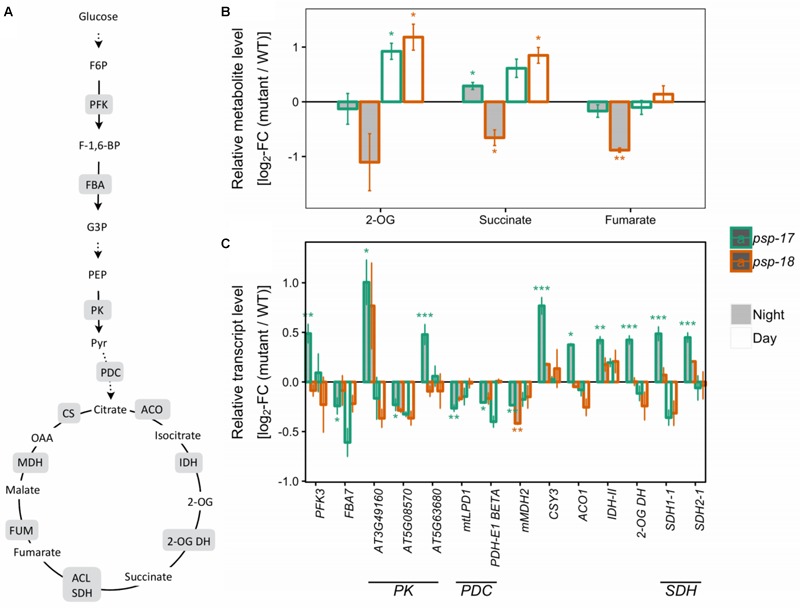
Glycolysis, the TCA cycle. **(A)** Schematic presentation (some steps omitted for clarity). **(B)** Metabolite amounts in *psp* mutants (*n* = 4, except *psp-18* night: *n* = 3) relative to the wt. Asterisks indicate significant differences between respective mutant and wt as determined by Student’s *t*-test (^∗^*p* < 0.05, ^∗∗^*p* < 0.01, and ^∗∗∗^*p* < 0.00). **(C)** Transcript abundances in *psp* mutants (*n* = 3, except *psp-18* night: *n* = 2) relative to the wt. Asterisks indicate differential abundance between respective mutant and wt as determined by Sleuth (^∗^FDR < 0.05, ^∗∗^FDR < 0.01, and ^∗∗∗^FDR < 0.001). Only significantly different data between either *psp* mutant and wt at either time point is shown. Additional transcripts and metabolites are presented in Supplementary Datasets S1, S2. 2-OG DH, 2-OG dehydrogenase, ACL, Succinate-CoA ligase; ACO, aconitase; CS, citrate synthase; FBA, fructose-bisphosphate aldolase; FUM, fumarase; IDH, isocitrate dehydrogenase; MDH, malate dehydrogenase; NAD-ME, NAD-malic enzyme; PDC, pyruvate dehydrogenase complex; PFK, phosphofructokinase; PK, pyruvate kinase; SDH, succinate dehydrogenase.

Several genes encoding specific enzymes of glycolysis had significantly higher transcript amounts at night in the *psp-17* line relative to the WT (Figure 3). The same trend was observed in the *psp-18* line, although less strong and not passing the significance threshold. Significant differences in TCA intermediates were observed for 2-oxoglutarate (2-OG), succinate, and fumarate that showed increased amounts at day and decreased amounts at night for both mutant lines compared to the WT (Figure 3).

**FIGURE 4:**
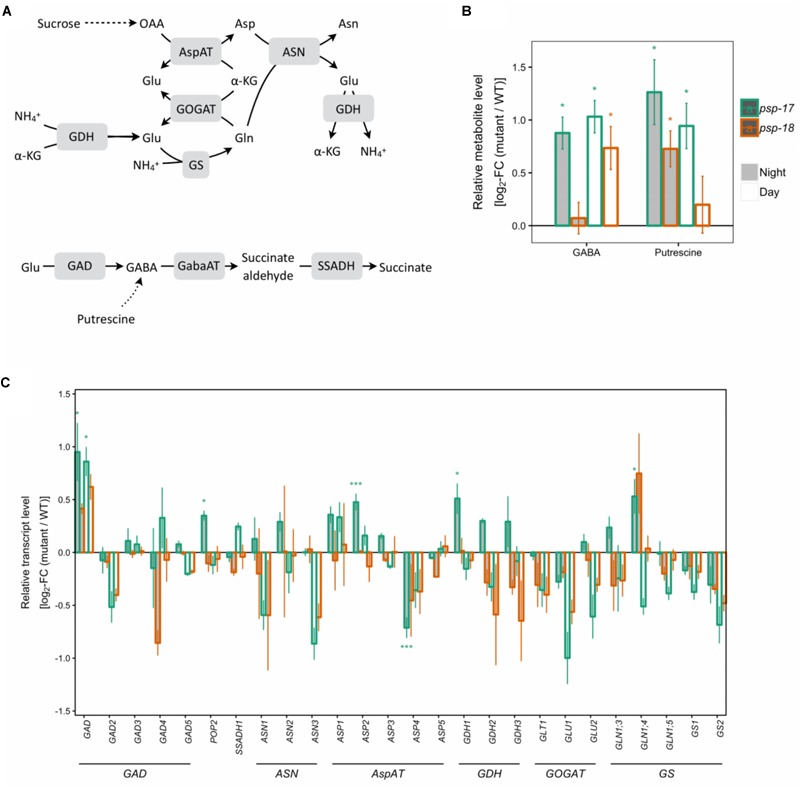
Nitrogen assimilation and the GABA shunt. **(A)** Schematic presentation. **(B)** Metabolite amounts in *psp* mutants (*n* = 4, except *psp-18* night: *n* = 3) relative to the wt. Asterisks indicate significant differences between respective mutant and wt as determined by Student’s *t*-test (^∗^*p* < 0.05, ^∗∗^*p* < 0.01, and ^∗∗∗^*p* < 0.00). **(C)** Transcript abundances in *psp* mutants (*n* = 3, except psp-18 night: *n* = 2) relative to the wt. Asterisks indicate differential abundance between respective mutant and wt as determined by Sleuth (^∗^FDR < 0.05, ^∗∗^FDR < 0.01, and ^∗∗∗^FDR < 0.001). AspAT, aspartate aminotransferase; ASN, Asparagine synthetase; GABA, gamma-Aminobutyric acid; GabaAT, GABA aminotransferase; GDH, glutamate dehydrogenase; GOGAT, glutamate synthase; GS, glutamine synthase; SSADH, Succinate semialdehyde dehydrogenase.

Transcripts of several key genes encoding enzymes of primary nitrogen assimilation were less abundant at day and night in both mutant lines with no statistically significant differences (Figure 4). A significant increase in transcript amount was observed for glutamate decarboxylase *GAD1* concomitant with increase in GABA content in *psp* line #17. The same trend for *GAD1* was observed for the *psp-18* mutant (Figure 4). In addition, an enhanced putrescine pool size was observed for both mutant lines compared to the WT (Figure 4).

Apart from changes in photorespiratory and TCA cycle intermediates, GABA and putrescine, out of 39 metabolites analyzed only branched-chain amino acids (BCAA), such as isoleucine (Ile), leucine (Leu), Valine (Val), and alanine (Ala), and further shikimate and threonine (Thr) showed accumulation at day and night in both mutants with significant differences observed for the *psp-17* line (Supplementary Figure S4). Surprisingly, most of the genes encoding transaminases of these amino acids displayed lower transcript amounts in both mutant lines compared to WT, with significant differences observed for *psp-17* line (Supplementary Dataset S3).

In summary, knock-down of *PSP* affected metabolome and transcriptome in similar ways in both mutant lines relative to the WT. Consistent with stronger repression of *PSP* gene expression, the observed effects were always stronger in the *psp-17* line compared to the *psp-18* line, which typically displayed similar trends as *psp-17* (Supplementary Figure S1B). To elucidate the importance of the phosphorylated pathway of Ser production for Cys synthesis under normal condition and upon Cd treatment, the *psp-17* line was chosen for further experiments.

### Sulfur Fluxes and Sulfur Metabolite Contents in *A. thaliana* WT and Its *PSP-17* Mutant Differ Between Day and Night and Are Affected by Cd Treatment

**FIGURE 5:**
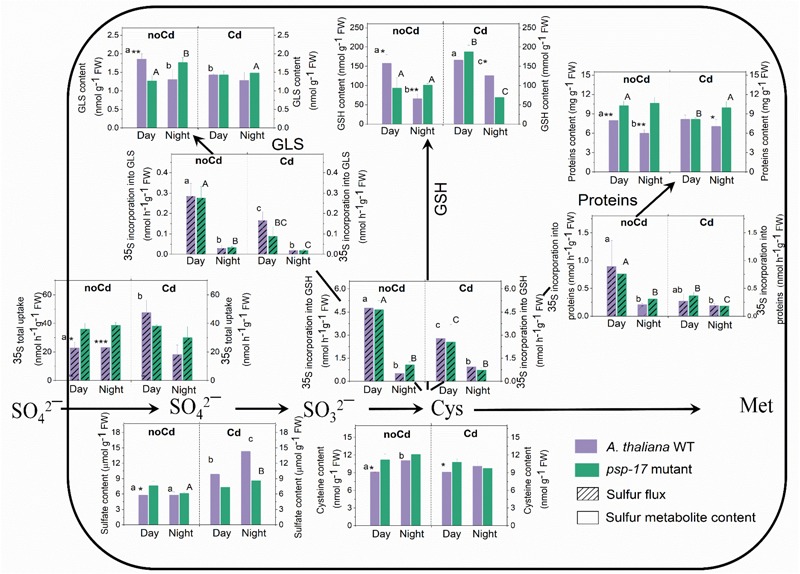
Foliar sulfur metabolite content and sulfur fluxes in the *A. thaliana* WT and the *psp-17* mutant at day and night and upon Cd exposure. Asterisks indicate significant differences between different plant types within same treatment determined by Student’s *t*-test (^∗^*P* < 0.05, ^∗∗^*P* < 0.01, and ^∗∗∗^*P* < 0.001). Small letters indicate significant differences between different treatments for WT plants determined by One-way Anova (*P* < 0.05). Capital letters indicate significant differences between different treatments for *psp-17* mutant determined by One-way Anova (*P* < 0.05). All values are means ± standard deviation of 3 replicates.

At day and night, sulfate uptake into the leaves was significantly higher in the *psp-17* mutant compared to the WT despite similar sulfate pool sizes. Despite similar sulfate uptake at night and day by both plant types (Figure 5), the flux of sulfate into proteins, GSH and GLS pools was strongly reduced at night compared to day for both, the WT and the *psp-17* mutant. The higher flux of sulfate into sulfur metabolites of the WT at day was accompanied by 1.4-, 2.4-, and 1.4-fold higher amounts of protein, GSH and GLS, respectively, compared to night exposure (Figure 5). Still the Cys pool was higher at night than at day in the WT. However, in the *psp-17* mutant, the amounts of protein, GSH, and GLS were significantly higher at night compared to day (Figure 5).

In the WT, Cd treatment at day enhanced the uptake of sulfate and the sulfate pool. Despite a lack of similar changes in sulfate uptake at night, the pool size of sulfate in the WT was higher upon Cd treatment compared to non-Cd control plants. Cd treatment did not significantly affect sulfate uptake and the pool size of sulfate in the mutant at both, day and night, except for a slight but significant increase in the pool size of sulfate in the mutant at night (Figure 5). Cd treatment negatively affected the flux of sulfur into proteins, GSH, and GLS at day and night for both plant types. Still Cd did not significantly affect the protein, Cys, and GLS pool sizes in the WT at either day or night exposure. However, the GSH content was almost twofold higher in the WT at night upon Cd treatment compared to non-Cd control plants. The *psp-17* mutant showed a significant decrease in protein and an increase in the GSH content at day upon Cd treatment, while the Cys and GLS contents did not show any significant changes. In addition, the Cd treatment significantly decreased the Cys and GSH pools in the *psp-17* mutant at night compared to non-Cd control plants (Figure 5).

The flux of sulfate into GSH, protein and GLS followed the same pattern for the WT and the *psp-17* mutant. At both, day and night, higher amounts of protein were observed in the *psp-17* mutant compared to the WT. In the *psp-17* mutant, the Cys pool was significantly higher during the day compared to the WT, while the GSH content was 1.6-fold reduced at day, but increased at night. A significant decrease at day and an increase at night were also observed for the GLS content in the *psp-17* mutant compared to the WT (Figure 5). Upon Cd treatment, significant differences were observed only for protein and GSH contents at night between the *psp-17* mutant and the WT (Figure 5). Apparently, Cd treatment abolished the differences between the plant types at day and night.

**FIGURE 6:**
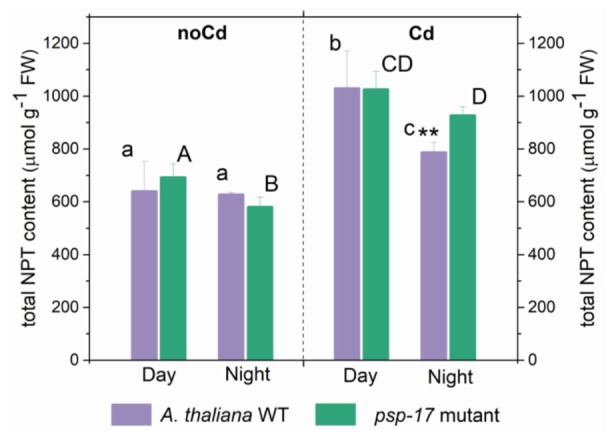
Foliar total non-protein thiols (NPT) content in A. *thaliana* WT plants and the *psp-17* mutant at day and night and upon Cd exposure. Asterisks indicate significant differences between different plant types within same treatment determined by Student’s *t*-test (^∗^*P* < 0.05, ^∗∗^*P* < 0.01 and ^∗∗∗^*P* < 0.001). Small letters indicate significant differences between different treatments for WT plants determined by One-way Anova (*P* < 0.05). Capital letters indicate significant differences between different treatments for *psp-17* mutant determined by One-way Anova (*P* < 0.05). All values are means ± standard deviation of 3 replicates.

Total non-protein thiol (NPT) contents were similar in the WT and the *psp-17* mutant at both day and night (Figure 6). A significant increase in total thiol content was observed upon Cd treatment in both plant types at day and night. Total NPT contents increased 1.6- and 1.5-fold at day and 1.3- and 1.6-fold at night in the WT and the *psp-17* mutant, respectively. At night, total NPT contents in the *psp-17* mutant were 1.2-fold higher upon Cd treatment compared to the WT (Figure 6).

### The Amino Acid Precursors of Thiol Metabolites Are Not Affected by the *PSP* Repression, Yet by Cd Treatment

**FIGURE 7:**
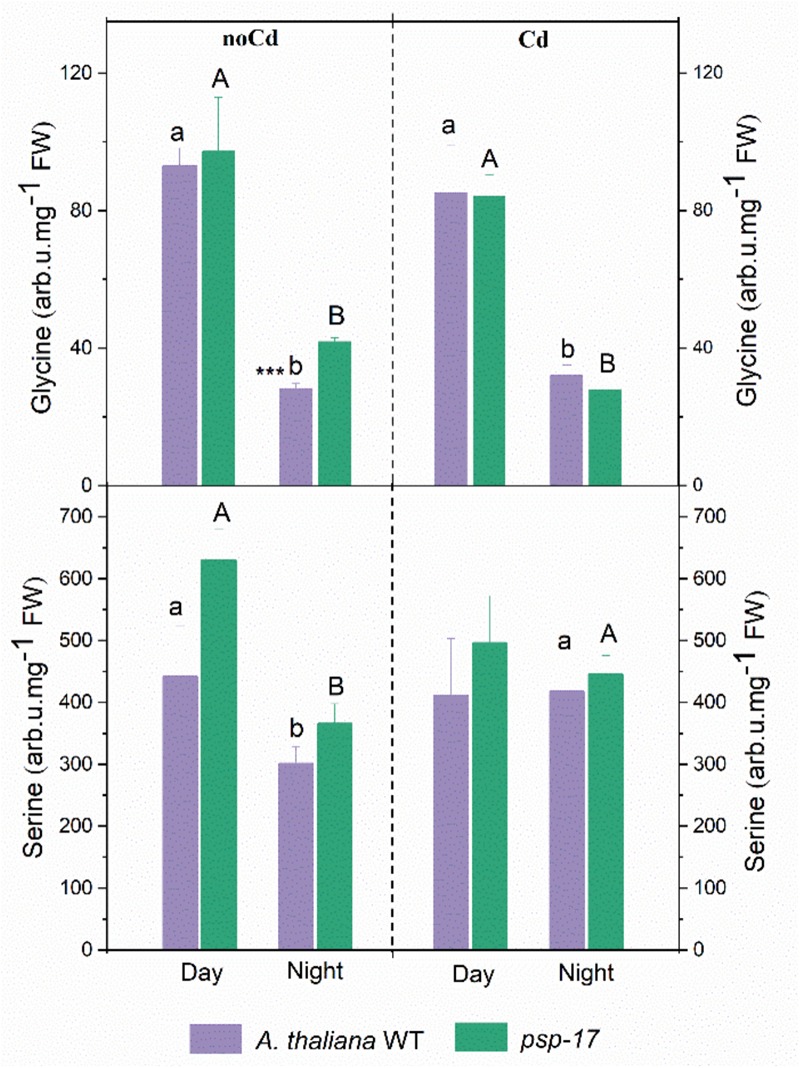
Amount of Gly and Ser in *A. thaliana* WT and *psp-17* mutant at day and night and upon Cd exposure. Asterisks indicate significant differences between different plant types within same treatment determined by Student’s *t*-test (^∗^*P* < 0.05, ^∗∗^*P* < 0.01 and ^∗∗∗^*P* < 0.001). Small letter indicate significant differences between different treatments for WT plants determined by One-way Anova (*P* < 0.05). Capital letters indicate significant differences between different treatments for *psp-17* mutant determined by One-way Anova (*P* < 0.05). All values are means ± standard deviation of 3 replicates.

The amount of Gly significantly decreased at night compared to day in controls and Cd-treated plants of both plant types (Figure 7). No differences in Gly content between the WT and the *psp-17* mutant were observed except for control plants at night. The Gly content in the *psp-17* mutant was significantly higher than in WT control plants at night. Upon Cd treatment at night, these differences were abolished between the two genotypes.

Ser contents were 1.5-fold lower in the WT and 1.7-fold lower in the *psp-17* mutant at night compared to the day. At night upon Cd treatment, the Ser content significantly increased in both genotypes compared to control plants, whereas it remained similar to control plants in the WT upon Cd treatment at day. No statistically significant differences in Ser contents were observed between the two genotypes in controls or Cd-treated plants at day or night.

### Primary Metabolite Abundances in the WT and Its *PSP-17* Mutant Differ Between Day and Night and Are Affected by Cd Treatment

Supplementary Figure S5 shows significant differences in primary metabolite abundances in WT plants and its *psp-17* mutant between day and night. Out of a total of 15 metabolites that changed between day and night in the *psp-17* mutant, 13 metabolites had higher abundances at day and only 2 had higher abundances at night (Supplementary Figure S5A). In WT plants, an equal number of metabolites had higher abundances at day and at night. Only two metabolites at day and one at night increased in both the WT and the *psp-17* mutant. Upon Cd treatment, twofold more metabolites showed enhanced abundances at day than at night (Supplementary Figure S5B). Also the number of metabolites with enhanced abundance at day and night shared between the *psp-17* mutant and WT increased upon Cd treatment. Five out of 7 metabolites in the WT and only one out of 6 in the *psp-17* mutant showed significantly increased abundances under Cd treatment at day (Supplementary Figure S5C). Two metabolites at day and 5 at day upon Cd treatment showed increased abundance in both the WT and the *psp-17* mutant. In the WT 10 out of total 16 metabolites showed significantly higher abundance at night upon Cd treatment compared to control plants (Supplementary Figure S5D). In the *psp-17* mutant, an equal number of metabolite had higher abundance at day and at night. Only one metabolite with higher abundance at night and 3 at night upon Cd were shared between the WT and the *psp-17* mutant.

### Some Sugars Together With Amino Acids Derived From Catabolic C Metabolism Are Differently Affected by Day/Night and Cd Treatment in WT and *PSP-17* Mutant

Changes in sugar amounts between day and night as well as control and Cd treated plants for both day and night were more significant in the psp-17 mutant than in WT plants (Supplementary Table S2). The amounts of sucrose at day, xylose at night and fructose and manose at both day and night, were higher in the psp-17 mutant compared to the WT (Supplementary Table S2). Cd treatment reduced the amounts of fructose (Fru) and mannose (Man) at day and of glucose (Glc), maltose (Mal), Man, and Xylose (Xyl) at night in the *psp-17* mutant (Supplementary Table S2).

**FIGURE 8:**
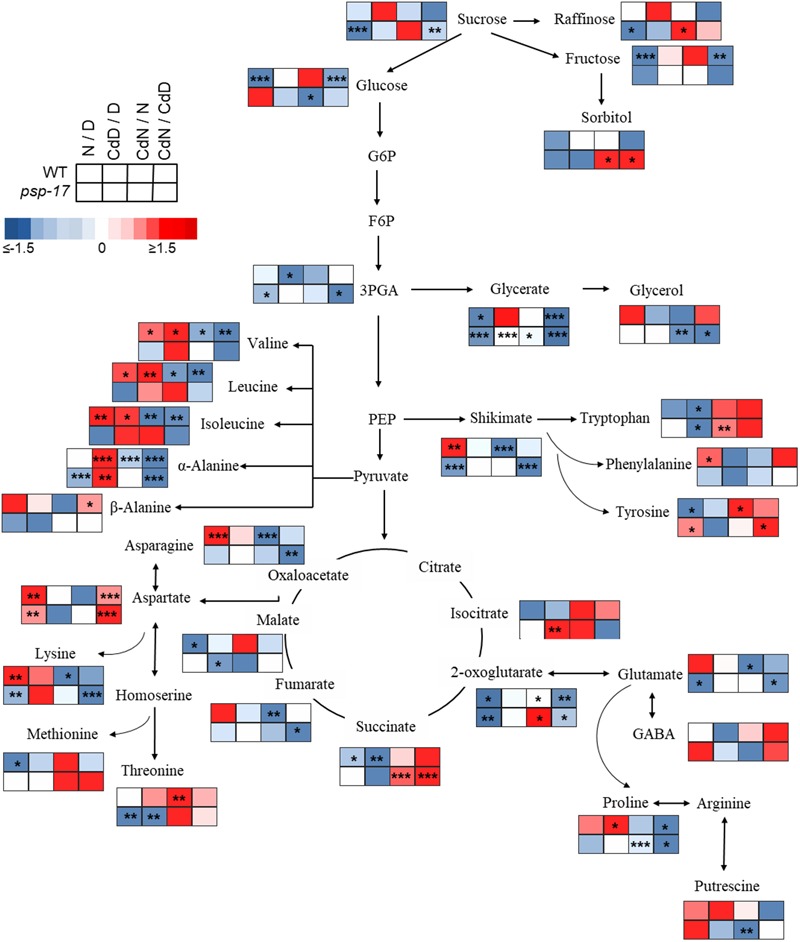
Schematic overview of primary metabolite abundance in *A. thaliana* WT and *psp-17* mutant at day and night and upon Cd treatment, (*n* = 3). Color code indicating log2-fold change (FC) of metabolites concentrations (blue-decreased, red-increased concentration). Tiles from right to left represent log2-fold change (FC) between night and day (N/D), Cd treated plants at day and control plants at day (CdD/D), Cd treated plants at night and control plants at night (CdN/N) and Cd treated plants at night and day (CdN/CdD). Upper four tiles represent log2-fold change (FC) for WT plants; lower four tile represent log2-fold change (FC) for *psp-17* mutant plants. Asterisks indicate significant differences between day and night and Cd treatment and control at day and night for each plant genotype determined by Student’s *t*-test (^∗^*P* < 0.05, ^∗∗^*P* < 0.01 and ^∗∗∗^*P* < 0.001).

**FIGURE 9:**
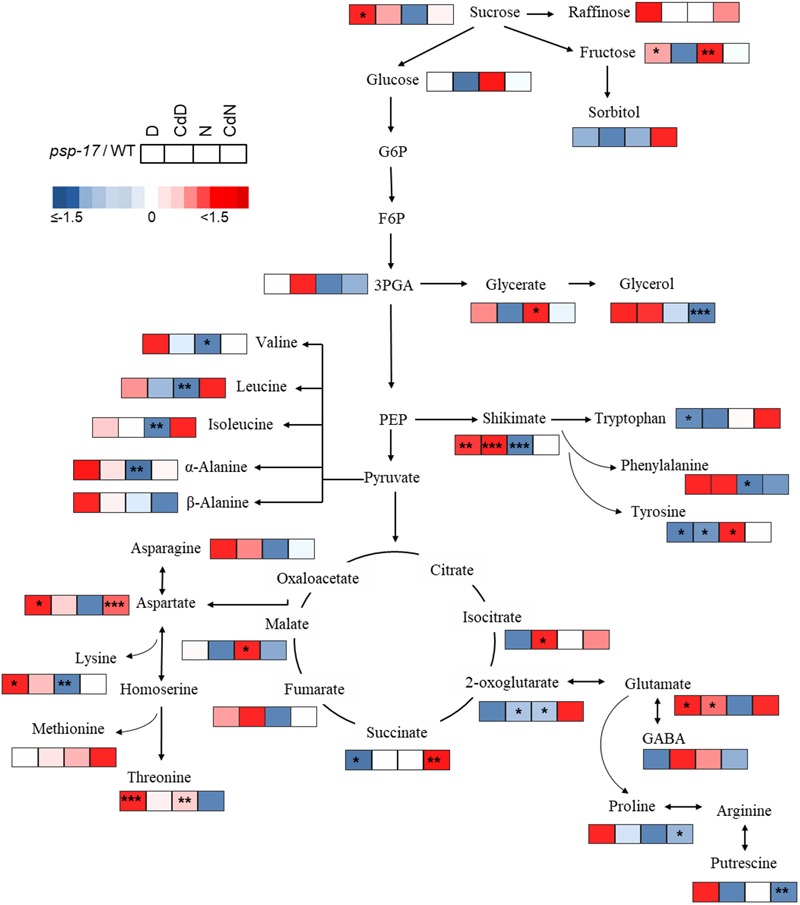
Schematic overview of carbon catabolic metabolism and its derived amino acids in *A. thaliana* WT and *psp-17* mutant at day and night and upon Cd treatment, (*n* = 3). Color code indicating log2-fold changes (FC) of metabolites concentrations (blue-decreased, red-increased concentration). Tiles from left to right represent log2-fold change (FC) between *psp-17* mutant and WT for day (D), Cd treated plants at day CdD), night (N) and Cd treated plants at night (CdN). Asterisks indicate significant differences between WT and *psp-17* mutant at day and night and upon Cd at day and night day and night determined by Student’s *t*-test (^∗^*P* < 0.05, ^∗∗^*P* < 0.01 and ^∗∗∗^*P* < 0.001).

Most significant changes between WT plants and the *psp-17* mutant at day/night and Cd treatment were observed for amino acid abundances (Figures 8, 9 and Supplementary Table S2). These changes were particularly observed for pyruvate derived amino acids in the WT, and for shikimate and its derived aromatic amino acids in both plant genotypes (Figures 8, 9 and Supplementary Table S2). Abundances of oxaloacetate derived amino acids such as asparagine (Asn), aspartate (Asp), lysine (Lys), were differently affected by day/night and by Cd treatment in WT and *psp-17* mutants.

Metabolite changes in WT plants and the *psp-17* mutant between day and night and between control and Cd-treated plants are shown in Figure 8. Pyruvate derived amino acids such as Valine (Val), Leucine (Le), and Isoleucine (Ile) were more affected by day/night and Cd treatment in the WT than in the *psp-17* mutant except for α-Alanine (α-Ala). In WT plants upon Cd treatment, the amount of pyruvate derived amino acids increased at day, but decreased at night compared to controls at day and night (Figure 8). The *psp-17* mutant showed a significant decrease in α-Ala at day compared to night (Figure 8). However, no changes in α-Ala were observed at night upon Cd treatment in the *psp-17* mutant. A significant decrease in Val, Leu, Ile, and Ala was observed at night in the *psp-17* mutant compared to the WT (Figure 9). Cd abolished these differences between the two genotypes at day and night.

In the WT, oxaloacetate derived amino acid such as Asp, Asn, and Lys showed a significant increase at night compared to day (Figure 8). Cd treatment significantly decreased the amount of Asn and Lys at night in WT plants (Figure 8). Asp contents were significantly increased, while Lys contents were decreased in the *psp-17* mutant at night compared to day. Cd did not significantly affect the amount of Asn, Asp, and Lys in the *psp-17* mutant at day or night.

Significant increases of Asp, Asn, and Lys contents were observed in the *psp-17* mutant at day compared to the WT (Figure 9). Upon Cd treatment at night, the Asp contents significantly increased in the *psp-17* mutant compared to the WT.

Shikimate, the main carbon donor for aromatic amino acids (Maeda and Dudareva, 2012), was increased in WT at night compared to the day. This increase abolished upon Cd treatment (Figure 8). The content of shikimate significantly decreased at night upon Cd treatment in WT plants. It was significantly lower in the *psp-17* mutant at night compared to the day. The *psp-17* mutant showed a significant increase in shikimate at day and at day upon Cd treatment compared to the WT (Figure 9). At night, shikimate contents significantly decreased in the *psp-17* mutant compared to the WT. However, Cd treatment diminished these differences between two genotypes at night.

Cd treatment did not affect the glutamate (Glu) content at day in the WT, while at night Glu contents significantly decreased upon Cd treatment. Different to the WT, Glu contents in the *psp-17* mutant were significantly lower at night compared to the day. Cd treatment did not significantly affect Glu contents at day and night in the *psp-17* mutant. Still, the Glu content was significantly higher in the *psp-17* mutant at day and at day upon Cd treatment compared to the WT (Figure 9).

The stress related amino acid proline (Pro) showed extreme changes upon Cd treatment in the WT. Cd treatment significantly increased Pro contents 208-fold in the WT at day, but decreased Pro contents 12-fold at night. In the *psp-17* mutant at night, Pro contents were 24-fold lower than at day. Cd treatment did not significantly affect Pro contents in the *psp-17* mutant at day, but decreased its content sixfold at night. At day, the Pro content was 143-fold higher in the *psp-17* mutant than in the WT (Figure 9). Cd treatment negatively affected Pro contents in the *psp-17* mutant compared to the WT, but this effect was significant only at night.

## Discussion

### The Mutant Lines *PSP-17* and *PSP-18* Show Similar Transcript and Metabolite Changes at Elevated CO_2_ That Differ From the *bou-2* Mutant Mostly in Intensity

In the photorespiratory *bou-2* mutant, the photorespiratory intermediate Gly was increased, whereas Ser plus glycerate decreased compared to WT at both elevated and ambient CO_2_ (Eisenhut et al., 2013). Accumulation of Gly and to lesser extend of Ser together with a decrease of transcript abundance of genes of the photorespiration pathway was observed in both the *psp-17* and the *psp-18* mutant line (Figure 1B and Supplementary Figure S3). This decrease may result from repressed photorespiration during growth at elevated CO_2_. However, knock-down of the *PSP* gene did not affect Ser contents in either mutant line, indicating that Ser synthesis was not regulated by transcriptional changes of genes of the photorespiratory pathway under the conditions applied. Both *psp* mutant lines showed a reduced transcript amount of key genes of nitrogen assimilation, also observed in the *bou-2* mutant, grown at elevated and shifted to ambient CO_2_ (Samuilov et al., 2018). In addition, similar to the *bou-2* mutant, an enhanced GABA pool size and *GAD1* transcript amount was observed at day and night for both *psp* mutant lines (Figure 4). Apparently, down-regulation of photorespiration and the phosphorylated pathway negatively affects nitrogen assimilation and GABA metabolism in a similar way. The processes involved in these metabolic changes require further attention. The shift from elevated to ambient CO_2_ resulted in an accumulation of amino acids such as Ala, Ile, Leu and Thr in the *bou-2* mutant (Eisenhut et al., 2013; Samuilov et al., 2018). Under the same growth conditions, also both *psp* mutant lines showed increased amounts of these amino acids at day and night (Supplementary Figure S4). However, this increase was less pronounced in the *psp* mutant lines compared to the *bou-2* mutant.

While the *bou-2* mutant showed increased transcript amounts of genes of the TCA cycle together with an accumulation of TCA intermediates, such as 2-OG and succinate at day and night (Samuilov et al., 2018), similar changes were not observed in the *psp-17* and *psp-18* mutant lines (Figure 3). Although transcript abundances of key genes of the CBBC in both mutant lines showed the same trend as previously observed in the *bou-2* mutant (Samuilov et al., 2018), the sugar content of *psp* mutants was much less affected compared to the *bou-2* mutant (Eisenhut et al., 2013; Samuilov et al., 2018) presumably due to higher transcript content of Rubisco subunit (*RBCL*) and an undisturbed TCA cycle. In both *psp* mutant lines the shikimate content was slightly increased at daytime compared to the night. Similar changes were not observed for the *bou-2* mutant (Eisenhut et al., 2013). In addition, the putrescine content was below the limit of detection in the *bou-2* mutant, while in both *psp* mutant lines its content was enhanced at day and night compared to WT plants.

Thus, when grown under the same conditions, *bou-2* and *psp* mutant lines mostly showed similar changes in photorespiratory metabolite and amino acid amounts and in transcript amounts of key enzymes of the photorespiratory pathway, primary nitrogen assimilation pathways and the Calvin Cycle (except for *RBCL* gene) relative to WT plants. However, these changes were more pronounced in the *bou-2* mutant due to reduced GDC activity than in the two *psp* mutant lines.

### Synthesis of Gly and Ser Are Not Affected by Knock-Down of the *PSP* Gene at Ambient CO_2_

Gly and Ser contents were higher at day than at night for both plant genotypes (Figure 7). Similar day/night fluctuations of Gly and Ser were observed in the study of Timm et al. (2013) in *Arabidopsis* plants with highest amounts at the end of the day and lowest amounts at the end of the night. Apparently, the *psp-17* mutant was not disturbed in Gly and Ser pool sizes. This result is consistent with the study of Benstein et al. (2013) in which the Ser concentration of an *Arabidopsis PGHD1*-silenced mutant was unaltered compared to control plants. By contrast, such a disturbance was observed with photorespiratory *Arabidopsis* mutants. Whereas overexpression of the H-protein of glycine decarboxylase (GDC) reduced glycine amounts and the Gly/Ser ratio (Timm et al., 2012), the *bou-2* knockout mutant showed reduced GDC activity and accumulated high amounts of Gly and Ser (Eisenhut et al., 2013; Samuilov et al., 2018). Therefore, the present study corroborates that the photorespiratory pathway is the main route of Ser synthesis in leaves at day. Still the *psp-17* mutant showed significantly higher amounts of Gly at night compared to the WT. Apparently, Gly synthesis at night exceeds its consumption.

The content of Gly and Ser upon Cd treatment has been reported to depend on plant species, the tissue examined, and Cd exposure time as well as concentration applied (Xie et al., 2014; Ma et al., 2017; Rabêlo et al., 2017). In the present study, Cd treatment did not significantly change foliar Gly and Ser contents of both plant types compared to control plants, except for Ser at night (Figure 7). Apparently, also Cd-dependent Ser synthesis of the *psp-17* mutant exceeds its consumption at night. These results with Cd treated and non-treated plants indicate a regulatory disorder of Gly and Ser synthesis in the leaves of the *psp-17* mutant at night.

### Knock-Down of the *PSP* Gene Does Not Disturb Sulfur Assimilation and Metabolism

Although sulfate uptake was similar at day and night, the incorporation of sulfate into GSH, GLS, and proteins was higher at day than at night for both plant genotypes, which is consistent with the results of earlier studies with *Arabidopsis* (Kopriva et al., 1999; Huseby et al., 2013; Samuilov et al., 2018). Adenosine 5’-phosphosulfate reductase (APR), which controls the flux through sulfate assimilation (Koprivova and Kopriva, 2014 and references therein), undergoes diurnal rhythm in plants grown under short day condition (Kopriva et al., 1999) with highest activity 2 h after light onset followed by increased SO_4_^2-^ uptake at day compared to night. However, in the study of Huseby et al. (2013) with *Arabidopsis* plants adapted to long day conditions, sulfate uptake was similar 4 h after light onset and 4 h before light. In addition, APR activity was highest during the first half of the day, while highest sulfate uptake was observed during the second half of the day. Therefore, similar sulfate uptake at day and night in the present experiments may be a consequence of growth conditions (12/12 h day/night) and/or harvesting time (day: 3 h after light onset; night: 5 h before light onset).

Similar to sulfur fluxes, also sulfur metabolite contents in WT leaves followed a day night regulation with higher amounts at day compared to night, except for the Cys pool (Figure 5). Accumulation of Cys at night could be a result of reduced nocturnal synthesis of GSH, GLS, and proteins. In the *psp-17* mutant, decreased expression of the *PSP* gene did not affect the day/night regulation of sulfur fluxes (Figure 5), but similar day/night fluctuations of sulfur metabolites were not observed. This result may be due to a slower turnover of sulfur metabolites in the *psp-17* mutant. Opposite results were obtained with the photorespiratory *bou-2* mutant that experienced heavily decreased sulfate uptake and fluxes at day, while sulfate incorporation into GSH and proteins did not follow day/night regulation (Samuilov et al., 2018). In addition, impaired photorespiration led to high accumulation of Cys and GSH in the *bou-2* mutant at both day and night. Compared to the *bou-2* mutant, in which sulfur metabolism was heavily disturbed by impaired photorespiration, decreased expression of the *PSP* gene of the phosphorylated pathway did not pose a strong negative effect on sulfur metabolism.

### Cd Treatment Negatively Affects the Sulfur Flux, but Enhances the Accumulation of Sulfur Metabolites

Incorporation of [^35^S]SO_4_^2-^ into GSH, GLS, and protein pools was reduced at day and night upon Cd treatment in both plant genotypes (Figure 5). Different to the present results with leaves, previous studies showed increased [^35^S]SO_4_^2-^ flux into roots upon Cd treatment (Nocito et al., 2006; Sun et al., 2007; Scheerer et al., 2010) by upregulation of a high-affinity sulfate transporter (Herbette et al., 2006). Apparently, roots – as the first target of Cd exposure – are of central significance for compensating the negative effects of Cd by detoxification *via* sulfur metabolism. Detoxification is thought to be mediated by an increased amount of total NPT and phytochelatins (PCs) in roots chelating the Cd taken up (Nocito et al., 2006; Sun et al., 2007; Jozefczak et al., 2014; Ferri et al., 2017). Thus, enhanced SO_4_^2-^ reduction and assimilation in the leaves in response to Cd will only be required, if Cd uptake exceeds the detoxification capacity of the roots and, as a consequence, Cd is transported to the leaves. This view is consistent with the observation that in plants grown under sufficient sulfate supply, Cd did not affect total GSH content in leaves (Sun et al., 2007; Ferri et al., 2017) or the response of foliar GSH content to Cd exposure was delayed (Jozefczak et al., 2014). However, to ensure sufficient supply of reduced sulfur for Cd detoxification in the roots, sulfate reduction and assimilation in the chloroplasts of the leaves and long-distance transport of GSH to the roots are required (Rennenberg and Herschbach, 2014; Luo et al., 2016). Previous studies observed an upregulation of genes of sulfate reduction and assimilation in response to Cd exposure (reviewed by Ernst et al., 2008). In addition, the transcription of the gene encoding the low-affinity sulfate transporters *Sultr2;1* that facilitates sulfate translocation from roots to shoots (Takahashi et al., 2011; Herschbach et al., 2012) was upregulated in response to Cd (Herbette et al., 2006). This upregulation could explain the enhanced sulfate pool size in WT plants upon Cd treatment observed at both day and night in the present study. It may be required to achieve enhanced foliar production of Cys and GSH for the allocation of thiols to roots upon Cd exposure.

In the *psp-17* mutant, sulfate content was largely unchanged upon Cd treatment, but was sufficient to maintain Cys synthesis for the enhanced demand of GSH (Figure 5). Compared to the WT, differences in GSH and protein contents were observed in the *psp-17* mutant upon Cd treatment. Apparently, in Cd-treated *psp-17* mutants the synthesis of GSH was favored over protein synthesis at day compared to the WT. Also the GLS amount decreased upon Cd treatment in the *psp-17* mutant, presumably to support the enhanced demand for GSH (Figure 5).

Phytochelatins constitute Cd- binding peptides that play important role in the detoxification of Cd. They are Cys-rich peptides synthesized from its precursor GSH by phytochelatin synthase (PCS) in the present of heavy metals (Gill and Tuteja, 2011 therein references). Therefore, total NPT were measured as they represent both, monothiols (e.g., Cys and GSH) and polythiols (phytochelatins) (Cobbett, 2000). In present study, the total NPT content was enhanced by Cd treatment at day and night in both plant types (Figure 6). These results are consistent with previous studies (Harada et al., 2002; Jozefczak et al., 2014; Ferri et al., 2017) attributing the enhanced foliar NPT contents upon Cd treatment to PCs and other thiol-peptides originating from foliar synthesis (Chen et al., 2006; Van Belleghem et al., 2007) and/or root-to-shoot transport (Gong et al., 2003).

Also the GLS pool sizes underwent significant changes in WT and *psp-17* mutant plants upon Cd treatment (Figure 5). Decreased GLS contents in both plant types upon Cd treatment are consistent with Cd induced downregulation of most genes involved in GLS synthesis observed in a study of Herbette et al. (2006) with *Arabidopsi*s plants, supporting the view of down-regulated GLS synthesis to support GSH production. Since GLS are sulfur containing secondary metabolites synthesized from Trp, Tyr, and Met and used in defense mechanism against mechanical injuries and herbivores (Halkier and Gershenzon, 2006), the present results indicate the distinct difference between defense strategies of *Arabidopsis* against Cd toxicity wounding / herbivore attack.

In the *psp-17* mutant the sulfur fluxes and sulfur metabolites show a similar pattern as WT plants at day and night upon Cd. In addition, Cd treatment did not disturb the day/night regulation of sulfur metabolism in both genotypes. Therefore, it can be concluded that the mutation of *PSP* gene did not significantly affect the response of sulfur metabolism to Cd treatment.

### Knock-Down of the *PSP* Gene Disturbs Nitrogen Metabolism

Different to the photorespiratory *bou*-*2* mutant (Eisenhut et al., 2013; Samuilov et al., 2018), the *psp-17* mutant did not show extreme changes in metabolite amounts. While the *Arabidopsis PSP* gene knock-down mutant was embryo lethal and *PGDH-1* silenced plants show the direct link between the phosphorylated pathway and ammonium assimilation, residual *PSP* gene expression in the *psp-17* mutant may have prevented strong effects on foliar. Still, amino acids derived from catabolic C metabolism were affected in the *psp-17* mutant. Apparently, accumulation of Suc and Fru at day lead to an accumulation of amino acids such as α-Ala, Asp, Lys and Glu in the *psp-17* mutant (Figure 8). Also the *Arabidopsis PGDH1*-silenced mutant showed an accumulation of amino acids such as Gln, Asp, and Ala (Benstein et al., 2013). These amino acids can provide ammonium for the synthesis of other amino acids and maintain the internal Glu amount (Hildebrandt et al., 2015). It has been suggested that Glu can be produced as a by-product of Lys metabolism (Galili et al., 2001; Stepansky et al., 2006); thus, the increased Lys production at day could contribute to the enhanced Glu amount in the *psp-17* mutant compared to the WT at day. The BCAA Val, Leu, and Ile experienced a significantly stronger decline in contents at night in *psp-17* mutant compared to WT, indicating an enhanced use of these amino acids for anabolic processes such as protein synthesis. This view is supported by significantly higher protein content of the *psp-17* mutant at both day and night compared to the WT. However, lower contents of BCAA could also result from catabolic reactions at night, such as the conversion to acetyl-CoA, propionyl-CoA and acetoacetate for energy generation (Joshi et al., 2010). In addition, it seems that a shift to preferential carbon flow into Shikimate and secondary metabolites over primary metabolism took place in the *psp-17* mutant compared to WT that also may have contributed to reduced BCAA amounts.

### Knock-Down of the *PSP* Gene Induces Additional Defense Mechanisms Against Cd Toxicity

Several studies showed that Cd stimulates the production of ROS in plants (Xu et al., 2009; He et al., 2015). Beside its role in PCs synthesis for heavy metal chelation, GSH plays important role in quenching ROS as a free radical scavenger and in the Foyer-Halliwell-Asada cycle (Noctor et al., 2012). In both genotypes, adjustment of metabolite pool sizes was observed as an adaptation to Cd exposure. WT plants showed extreme Pro accumulation upon Cd treatment as also observed in previous studies (Xu et al., 2009; reviewed by Gill et al., 2014). Pro was proposed as an effective ROS scavenger through the operation of the proline-cycle (Signorelli et al., 2013) and as direct metal binding chelator (reviewed by Anjum et al., 2014). In a study with *Solanum nigrum* under Cd stress Xu et al. (2009) observed that higher Pro amounts concomitantly enhanced the GSH content and the activity of antioxidative enzymes. Siripornadulsil et al. (2002) in a study using transgenic algae expressing Δ1-pyrroline-5-carboxylate synthetase (P5CS) showed that Pro more likely acts as an antioxidant in Cd-stressed cells, while GSH facilitate synthesis of PCs. Also, in the present study Pro presumably could have been used to scavenge ROS or as osmoprotectant to stabilize proteins and membranes (Shahjee et al., 2002) upon Cd stress. Due to the function of Pro in defense mechanism against Cd, similar contents of GSH in Cd-treated and control plants of the WT may have been sufficient to sustain PC synthesis and to prevent Cd toxicity at both day and night.

The *psp-17* mutant did not show an extreme increase in Pro content upon Cd treatment at day like the WT (Supplementary Table S2). Apparently, in the *psp-17* mutant GSH plays a primary role in detoxification of Cd. However, in addition to enhanced GSH content accumulation of Shikimate was observed in *psp-17* mutant upon Cd exposure at day (Figures 8, 9 and Supplementary Table S2). The increase in Shikimate content could have improved scavenging of ROS by phenolic compounds in *psp-17* mutant (Kısa et al., 2016; Mongkhonsin et al., 2016).

In addition, all pyruvate-derived amino acids analyzed significantly increased in the WT, while in the *psp-17* mutant only Ala was significantly enhanced upon Cd treatment (Figure 8 and Supplementary Table S2). Similar results were observed in previous studies with *Arabidopsis* exposed to Cd (Sun et al., 2010; Keunen et al., 2016). Cd decreased the Asp and Asn content, while it did not affect the Glu content in both genotypes (Figure 8 and Supplementary Table S2). This might be a buffering effect of enhanced Ala accumulation in both plant genotypes and, additionally, of enhanced Pro in the WT, since Ala and Pro are direct products of glutamic acid metabolism. Ala can be directly produced from pyruvate thereby converting 2-OG to Glu by alanine amino transferase (Hildebrandt et al., 2015). Pro is synthesized directly from Glu by a set of three enzymatic reactions (Sharma and Dietz, 2006). In the present study, it seems that Ala and Pro are used as ammonium sink rather than Glu, Asp and/or Asn in response to Cd stress (Gouia et al., 2003; Nikiforova et al., 2006).

## Conclusion

In this work, repression of the phosphoserine phosphatase, was chosen to elucidate the importance of the phosphorylated pathway of Ser production for Cys synthesis. Compared to photorespiratory impaired mutants, *PSP* knock-down did not negatively affect Gly and Ser content at day, while Gly accumulated in *psp-17* mutant at night, presumably due to its lower consumption. Analysis of [^35^S]SO_4_^2-^ flux showed that reduced amounts of *PSP* transcript did not significantly disturb sulfur fluxes into S-rich metabolites that followed day/night regulation. However, pool size of Cys, GSH, and proteins was similar at day and night, probably due to their slower turnover in *psp-17* mutant. Apparently, Ser provision for synthesis of Cys and other S-rich metabolites was not altered in *psp-17* mutant due to knock-down in *PSP* gene. The present results confirmed that the phosphorylated pathway plays a minor role in Ser provision for Cys synthesis at night and day in photosynthetic tissue, since down-regulation of *psp* transcript abundance did not affect (or only slightly affected) the cellular Cys level (hypothesis 2 rejected). Therefore, it is suggested that photorespiratory produced Ser is the main source for Cys biosynthesis in photosynthetic tissue (which supports hypothesis 1). Cd did not affect foliar GSH content of WT presumably due to high detoxification capacity of the roots to chelate Cd while synthesis of GSH in the *psp-17* mutant upon Cd treatment was favored over protein and GLS synthesis (which is similar to hypothesis 3). Observed higher content of NPT in both genotypes could be the result of PCs and other thiol-peptides originating from root-to shoot transport in WT and/or foliar synthesis in *psp-17* mutant. The observed changes in sulfur metabolism could be the result of an adjustment in amino acid pool sizes as an adaptation to Cd exposure. Accumulation of Pro in WT and shikimate precursors of many phenolic compounds in *psp-17* mutant could serve as ROS scavengers that enable GSH to be used primarily as Cd chelation. In the present study it seems that two genotypes employ different strategies for Cd detoxification. For better understanding of phosphorylated Ser biosynthesis for Cys synthesis in non-photosynthetic tissue and its significance of Ser supply for Cys synthesis upon Cd exposure further experiments with roots should be considered in the future.

## Author Contributions

HR and AW conceived the experiments. SS and NR designed the experiments. SS, NR, and SF performed the experiments. SS and DB analyzed data and generated figures and tables. SK, TM-A, LA, NR, and SF contributed reagents, materials, and analytical tools. SS and HR wrote the manuscript. AW, SK, and DB commented on the manuscript.

## Conflict of Interest Statement

The authors declare that the research was conducted in the absence of any commercial or financial relationships that could be construed as a potential conflict of interest.

## Footnotes

1 http://www.bioinformatics.babraham.ac.uk/projects/fastqc/

2 http://www.arabidopsis.org/

3 http://www.R-project.org

4 http://bioinformatics.psb.ugent.be/webtools/Venn/

5 https://www.ncbi.nlm.nih.gov/geo/query/acc.cgi?&acc=GSE112254
