# Corrigendum: Knock-Down of the Phosphoserine Phosphatase Gene Effects Rather N- Than S-Metabolism in *Arabidopsis thaliana*

**DOI:** 10.3389/fpls.2019.00325

**Published:** 2019-03-26

**Authors:** Sladjana Samuilov, Nadine Rademacher, Dominik Brilhaus, Samantha Flachbart, Leila Arab, Stanislav Kopriva, Andreas P. M. Weber, Tabea Mettler-Altmann, Heinz Rennenberg

**Affiliations:** ^1^Chair of Tree Physiology, Institute of Forest Sciences, Faculty of Environment and Natural Resources, University of Freiburg, Freiburg, Germany; ^2^Institute of Plant Biochemistry, Cluster of Excellence on Plant Sciences, Heinrich Heine University, Düsseldorf, Germany; ^3^Botanical Institute, Cluster of Excellence on Plant Sciences, University of Cologne, Cologne, Germany; ^4^College of Science, King Saud University, Riyadh, Saudi Arabia

**Keywords:** amino acids, Cd treatment, cysteine, glutathione, glycine, phosphorylated pathway, serine

In the original article, there was a mistake in Figure 6 as published. On the y-axis “nmol g^−1^ FW” was used instead of “μmol g^−1^ FW” for the total NPT content measurement. The corrected Figure 6 appears below.

**Figure 6:**
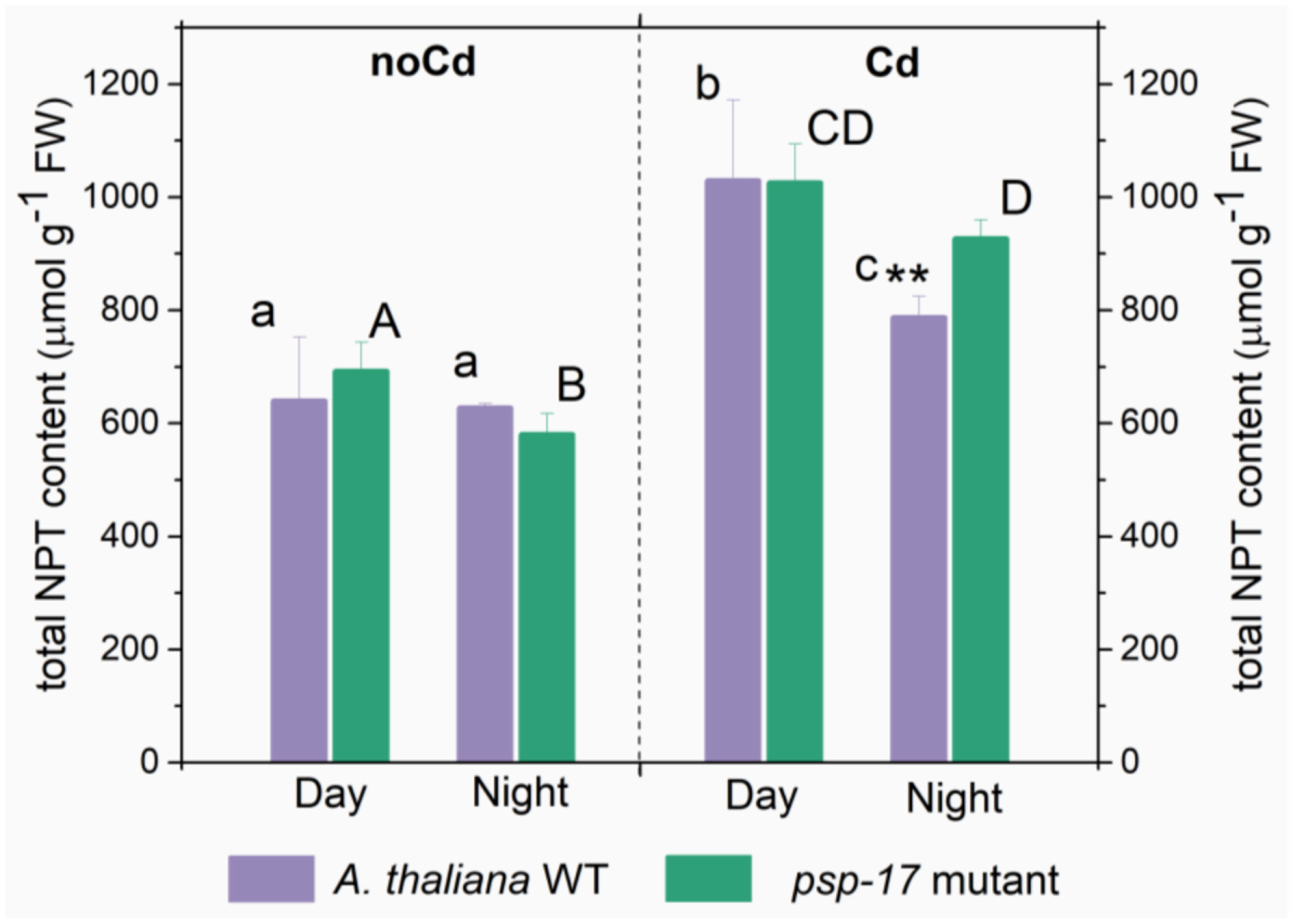
Foliar total non-protein thiols (NPT) content in *A. thaliana* WT plants and the *psp-17* mutant at day and night and upon Cd exposure. Asterisks indicate significant differences between different plant types within same treatment determined by Student's t-test (^*^P < 0.05, ^**^P < 0.01 and ^***^P < 0.001). Small letters indicate significant differences between different treatments for WT plants determined by One-way Anova (*P* < 0.05). Capital letters indicate significant differences between different treatments for *psp-17* mutant determined by One-way Anova (*P* < 0.05). All values are means ± standard deviation of 3 replicates.

A correction has been made to the Materials and Methods, subsection Quantification of Total Non-Protein Thiols:

“For the determination of total non-protein thiols (NPT), a modified method of Queval and Noctor (2007) was applied. Total thiols in leaf extract were assayed as 5,5′-dithio-bis-[2-nitrobenzoic acid]-reactive thiols (DTNB-reactive thiols) by spectrophotometry (Beckman UV- DU650, Beckman Coulter, United States) using glutathione (GSH) as a standard. Approximately 100 mg frozen leaf powder was extracted in 1 ml 0.2N HCl. Aliquots of 0.5 ml supernatant were transferred into fresh micro tubes (Sarstedt AG & Co., Nümbrecht, Germany) and neutralized with 0.4 ml 0.2 M NaOH in the presence of 50 μl 0.2 M NaH_2_PO_4_ (pH 5.6). For thiol quantification by spectrophotometry, each semi-micro cuvette (Sarstedt AG & Co., Nümbrecht, Germany) contained 500 μl phosphate-EDTA buffer (0.2 M NaH_2_PO_4_, pH 7.5; 10 mM EDTA), 50 μl of 12 mM DTNB and 450 μl neutralized sample extract (total volume 1 ml). For standards, the extract was replaced by 450 ml of 0, 10, 20, 30, 40, 50 μmol GSH. The absorbance was measured at a wavelength of 412 nm 3 min after addition of extract or standard.”

The authors apologize for these errors and state that they do not change the scientific conclusions of the article in any way. The original article has been updated.
