# Supplementary material for: Knock-Down of the Phosphoserine Phosphatase Gene Effects Rather N- Than S-Metabolism in Arabidopsis thaliana
Source: Front Plant Sci. 2018 Dec 11;9:1830. doi: 10.3389/fpls.2018.01830 (PMC6297848; doi:10.3389/fpls.2018.01830)
Supplement: Supplementary file 2 [file Data_Sheet_2.pdf]

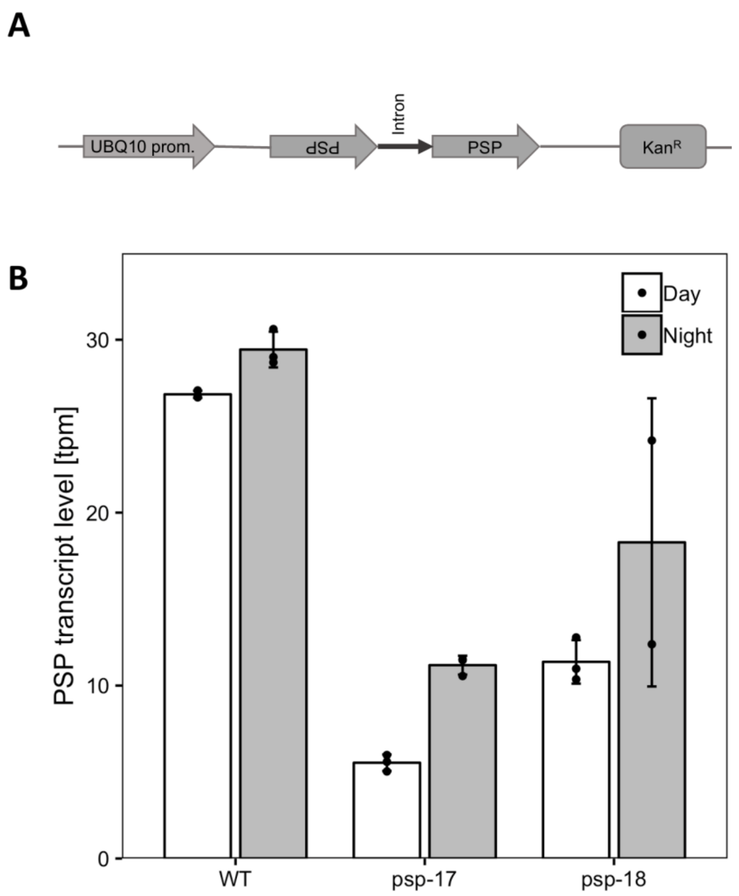

Supplemental Fig S1. Generation of *psp* mutants. A, Schematic diagram of the *PSP* RNAi construct. pSK-int vector was used as intermediated vector for cloning containing third intron of *Arabidopsis* Actin gene 11 (AtU27981, nt 1957-2111-gDNA) (Guo et al., 2003). DNA fragments encoding for sense and antisense *PSP* gene (At1g18640) were cloned in front respectively behind the intron. Complete construct is under the control of a Ubiquitin10 promoter. Kanamycin resistance gene was used as selection marker. **B,** Levels of transcript encoding *PSP* (At1g18640) in the wildtype and the two *psp* mutants. tpm, transcripts per million.

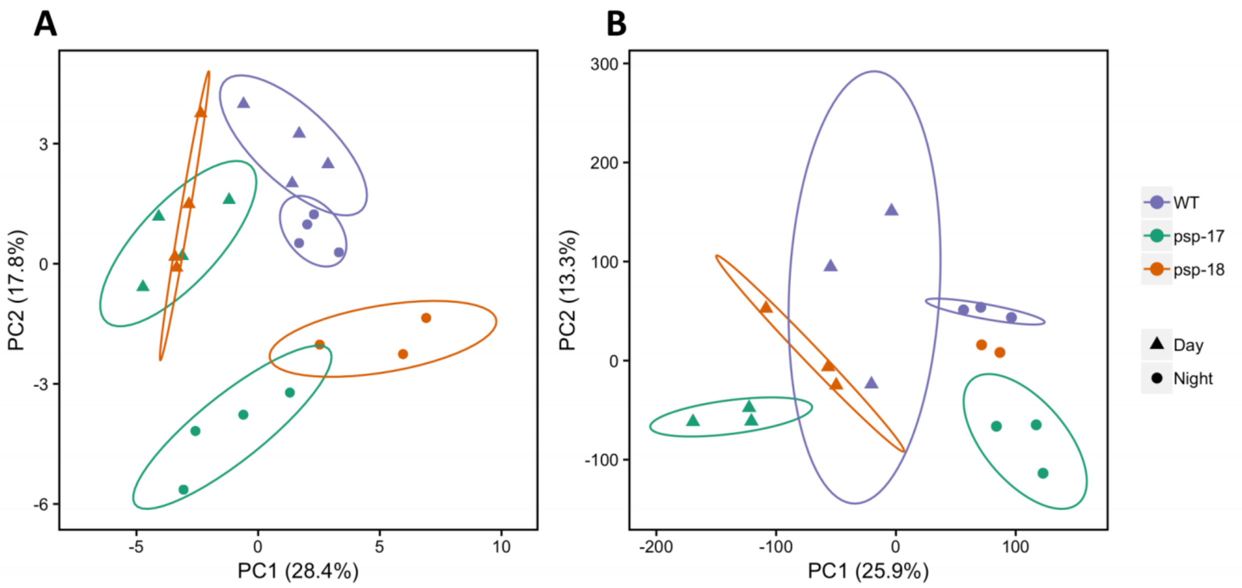

Supplemental Fig S2. Differences between the leaf metabolomes (**A**) and transcriptomes (**B**) of WT (purple), *psp-17* (green) and *psp-18* (orange) plants. Shown are the first two components of principal component analysis (PCA) of relative metabolite levels (**A**) or transcript abundances (**B**).

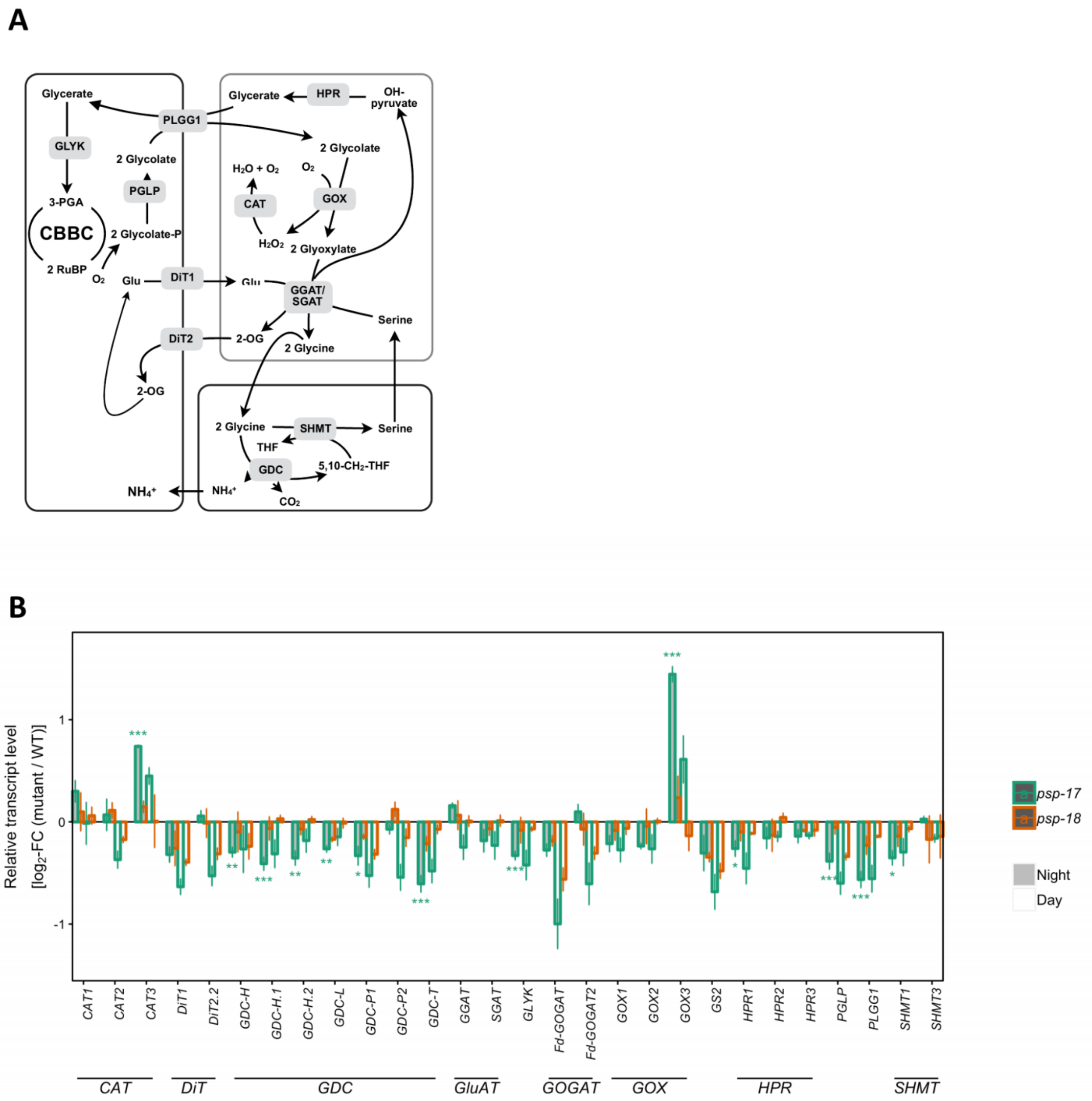

Supplemental Fig S3. Photorespiration. A, Schematic presentation. **B**, Transcript abundances in *psp* mutants (n = 3, except *psp-18* night: n = 2) relative to the wt. Asterisks indicate differential abundance between respective mutant and wt as determined by Sleuth (* FDR < 0.05, ** FDR < 0.01, and *** FDR < 0.001). 2-OG, 2-oxoglutarate; Glu, glutamate; THF, tetrahydrofolate; CAT, catalase; DiT, dicarboxylate transporter; GDC, glycine decarboxylase; GGAT, glutamate:glyoxylate aminotransferase; GLYK, glycerate kinase; GOX, Glycolate oxidase; HPR, peroxisomal hydroxypyruvate reductase; PGLP, Phosphoglycolate phosphatase; PLGG, plastidic glycolate glycerate transporter; SGAT, Serine-glutamate aminotransferase; SHMT, serine transhydroxymethyltransferase.

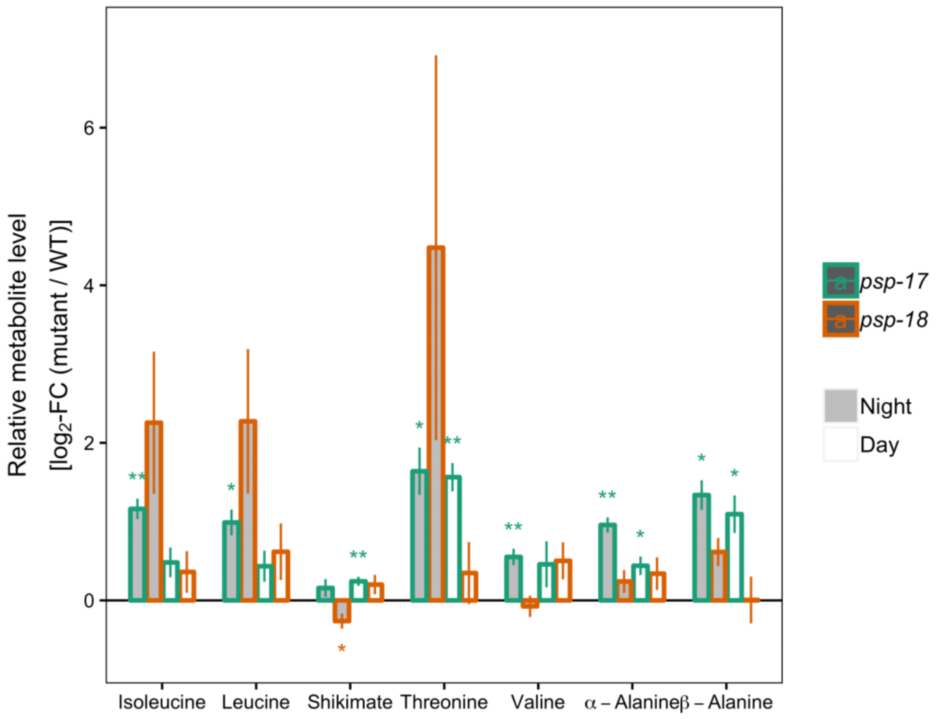

Supplemental Figure S4. Amino acid levels in *psp* mutants (n = 4, except *psp-18* night: n = 3) relative to the wt. Asterisks indicate significantly differences between respective mutant and wt as determined by Student's t-test (* p < 0.05, ** p < 0.01, and *** p < 0.00). Only significantly different data between either *psp* mutant and wt at either time point is shown. Additional metabolites are presented in Supplemental Datasets 2 and Fig. 4.

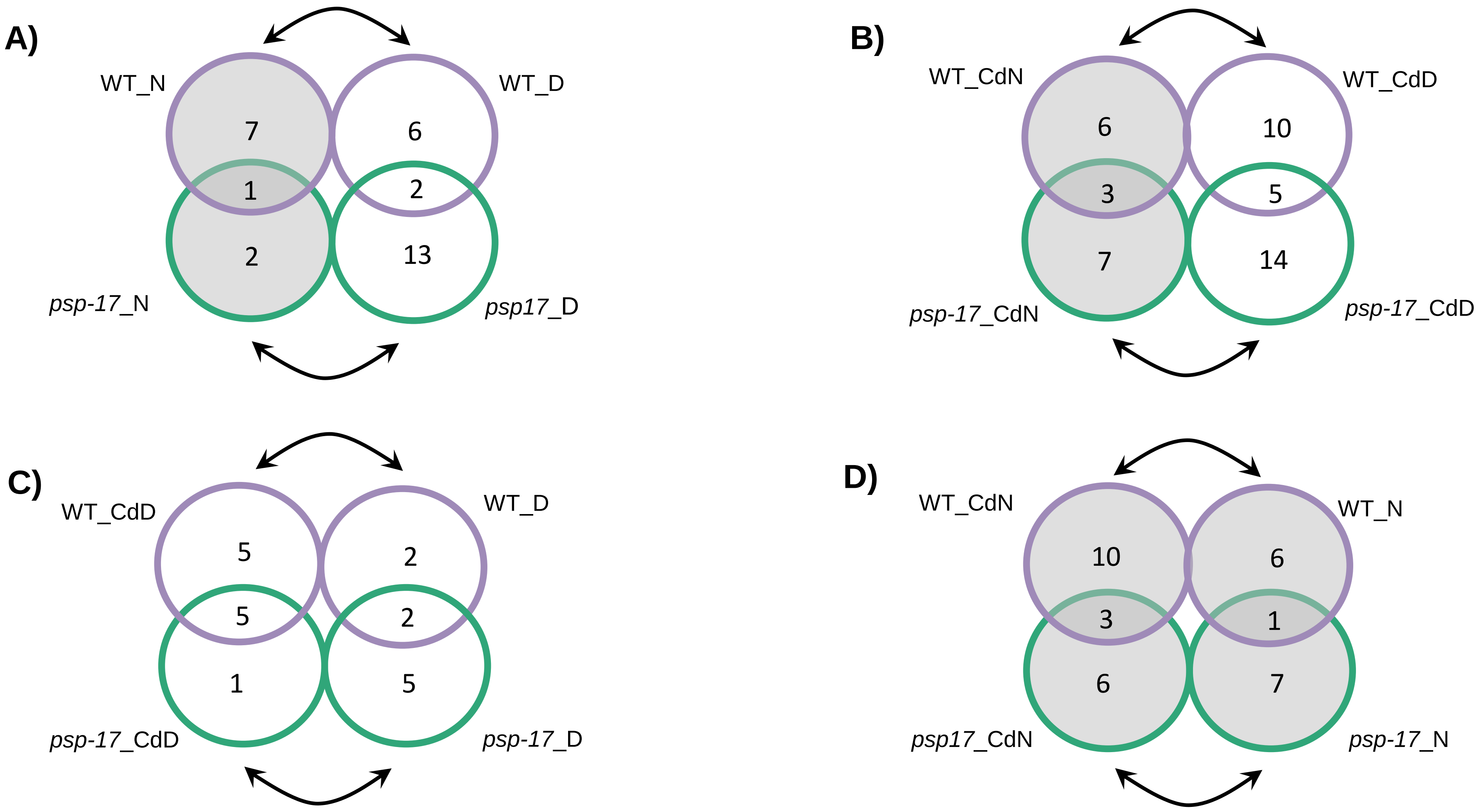

Supplemental Fig. S5. Venn diagrams represent only metabolite with higher abundances for each genotype between different time points (D, N) and treatments (CdD, CdN), (Arrows indicate the comparison direction) as well as higher metabolite abundances shared between genotypes at the same time point and treatments. Purple circles indicate *A. thaliana* WT plants; green circles indicate *psp-17* mutant plants; Grey coloured circles represent night; empty circles represent day.
