# Supplementary material for: Knock-Down of the Phosphoserine Phosphatase Gene Effects Rather N- Than S-Metabolism in Arabidopsis thaliana
Source: Front Plant Sci. 2018 Dec 11;9:1830. doi: 10.3389/fpls.2018.01830 (PMC6297848; doi:10.3389/fpls.2018.01830)
Supplement: Supplementary file 3 [file Data_Sheet_3.pdf]

**Suppl. Table S1.** Oligonucleotides used in this study for generation and selection of PSP knockdown mutant plants.

| Primer name | Description | Sequence | Restriction sites |
|---|---|---|---|
| P1 | PSP-AS-1-3'-5'-fwd | ccaGGGCCCcacCAATGTTATTGGCTCTGAGTTTCT | ApaI |
| P2 | PSP-AS-1-3'-5'-rev | ccaAAGCTTccaGACTTCACCAACCAACAAACAC | HindIII |
| P3 | PSP-AS-1-5'-3'-fwd | ccaGAATTCccaGACTTCACCAACCAACAAACA | EcoRI |
| P4 | PSP-AS-1-5'-3'-rev | ccaACTAGTccaCAATGTTATTGGCTCTGAGTTTCT | SpeI |
| P5 | Ubiquitin10-prom-screen-fwd | CGCCTTAGCTTTCTCGTGAC | |
| P6 | PSP-screen-rev | TCTCGCGCTCCAAGTTTAAT | |
| P7 | PSP-RT-fwd | CCATGATTGGAGATGGTGCT | |
| P8 | PSP-RT-rev | GGCTTCACGAAGCTGAACTC | |

**Suppl. Table S2.** Metabolite contents present in arbitrary units (arb.u. $mg^{-1}$ FW) in leaves of *Arabidopsis thaliana* WT and the *psp-17* mutant at day and night and upon Cd treatment at day and night (Day 3h after LO; Night 5h before LO). Asterisks indicate significant differences between different plant types within same treatment determined by Student's *t*-test (* P < 0.05, ** P < 0.01 and *** P < 0.001). Small letter indicate significant differences between different treatments for WT plants an dpsp17 mutant determined by One-way Anova (P < 0.05). All values are means ± standard deviation of 3 replicates.

| | | ***A. thaliana WT*** | | | | ***PSP-17* mutant** | | | | |
|---|---|---|---|---|---|---|---|---|---|---|
| | | **D** | **CdD** | **N** | **CdN** | **D** | **CdD** | **N** | **CdN** | ***T-test: WT vs PSP-17*** |
| **Sugars** | Fructose | 284.19±11.01 | 294.67±52.61 | 51.18±11.69 | 59.06±8.36 | 413.166±53.49a | 225.117±23.76b | 108.537±14.46c | 55.621±30.57c | T(D)*; T(N)** |
| | Glucose | 507.72±50.68a | 423.71±74.36a | 126.82±13.67b | 141.34±9.40b | 482.22±151.08a | 242.031±23.20ab | 497.799±140.61a | 129.154±75.75b | |
| | Maltose | 19.19±0.42a | 21.56±0.41ac | 30.01±3.10bc | 25.87±1.51c | 33.412±2.21a | 30.467±2.84ab | 37.016±3.61a | 30.675±1.17b | T(CdD)**; T(CdN)* |
| | Manose | 5.82±0.8 | 6.95±1.415 | 8.09±1.446 | 7.07±0.586 | 9.446±1.33a | 5.693±0.61b | 11.402±1.44a | 6.858±0.80b | T(D)*; T(N)** |
| | Raffinose | 42.51±7.644 | 62.90±15.958 | 41.46±13.01 | 43.39±17.293 | 228.79±116.73a | 79.103±19.68a | 45.121±4.75b | 113.056±40.48ab | |
| | Sorbitol | 1.41±0.383 | 1.36±0.279 | 1.16±0.334 | 1.09±0.202 | 0.941±0.18ab | 0.768±0.04a | 0.774±0.14a | 1.506±0.43b | |
| | Sucrose | 3633.97±645.85 | 3992.68±822.66 | 3677.55±532.19 | 3307.77±394.32 | 5827.285±1069.70a | 4724.32±528.97ab | 3374.286±529.69bc | 3616.695±177.14c | T(D)* |
| | Xylose | 16.73±3.27a | 14.25±0.63a | 8.09±1.30b | 18.21±0.59a | 11.07±1.60a | 11.369±0.80a | 18.856±2.45b | 13.128±0.82a | T(CdD)*; T(N)**; T(CdN)*** |
| **Acids involved in TCA cycle** | 2-oxaloglutarate | 16.11±4.56a | 11.90±1.78a | 6.68±0.46ab | 5.53±0.52b | 9.55±1.56a | 8.73±0.98a | 4.94±0.45b | 6.25±0.59c | |
| | Fumaric acid | 5438.62±706.69a | 4754.45±1115.62ab | 6778.01±901.17a | 4688.83±368.721b | 6267.533±822.92a | 6221.515±698.00a | 5655.141±628.60ab | 4817.739±679.193b | |
| | Isocitric acid | 785.30±217.87 | 679.05±109.06 | 632.25±124.82 | 775.30±109.33 | 631.054±43.66 | 957.928±133.83 | 632.228±177.98 | 955.998±271.99 | T(CdD)* |
| | Malic acid | 684.95±56.82a | 607.68±143.15ab | 456.96±46.18b | 509.56±49.34ab | 722.69±92.779 | 564.087±34.237 | 627.995±79.465 | 481.391±80.551 | T(N)* |
| | Proline | 1.13±0.59ac | 208.82±84.78b | 35.29±23.74abc | 3.38±1.42c | 143.173±128.38a | 104.843±40.75a | 6.232±1.12a | 1.025±0.22b | |
| | Succinic acid | 89.64±9.76a | 60.14±11.57b | 71.10±7.91ab | 76.67±9.53b | 66.482±8.57a | 62.105±6.46a | 72.591±9.96a | 123.154±18.19b | T(D)*; T(CdN)** |
| | 3PGA | 2.38±0.335 | 1.85±0.252 | 1.98±0.378 | 1.58±0.288 | 2.43±0.27a | 2.161±0.20a | 1.855±0.29ab | 1.51±0.34b | |
| **Secondary metabolites** | Glycolic acid | 25.91±3.74a | 25.87±4.15a | 18.27±0.98a | 29.34±3.30b | 24.035±3.965 | 18.033±2.411 | 25.187±0.893 | 31.248±11.036 | T(CdD)*; T(N)*** |
| | Shikimate | 44.34±7.59a | 37.62±4.59a | 59.55±4.72b | 30.41±4.03a | 68.567±5.13a | 60.342±3.75a | 36.739±5.87b | 29.67±5.69b | T(D)*; T(CdD)***; T(N)*** |
| **Amino acids** | Alpha alanine | 323.30±30.526a | 536.04±16.399b | 312.86±25.33a | 166.51±27.916c | 421.283±82.75a | 566.946±15.57c | 183.002±21.86b | 172.607±20.62b | T(D)*; T(CdD)* |
| | Aspartic acid | 381.23±54.46a | 391.05±29.01a | 734.88±171.96b | 568.95±10.84b | 543.959±60.00a | 439.13±46.73a | 731.121±37.03b | 744.456±7.16b | T(D)*; T(CdN)*** |
| | Asparagine | 14.07±4.12a | 20.15±4.73a | 47.89±1.84b | 10.46±4.05a | 38.262±18.74ab | 37.532±4.45a | 17.928±3.44b | 8.063±5.63bc | T(CdD)**; T(N)*** |
| | Beta alanine | 3.46±0.607 | 3.63±0.158 | 4.26±1.058 | 4.06±0.0983 | 4.961±2.297 | 3.905±0.19 | 3.941±0.406 | 3.268±0.677 | |
| | GABA | 28.70±4.933 | 20.14±2.822 | 27.66±1.368 | 32.54±11.968 | 26.563±4.369 | 26.078±4.291 | 32.178±6.611 | 30.765±18.606 | |
| | Glutamic acid | 800.22±131.10ab | 829.13±142.38ab | 990.36±181.974a | 531.78±236.614b | 1154.641±225.62a | 1078.212±129.95a | 786.22±50.50b | 761.22 ±131.55b | T(D)* |
| | Glycine | 92.82±5.34a | 85.20±13.71a | 28.26±1.42b | 32.06±2.98b | 97.31±15.60a | 84.187±6.28a | 41.938±1.07b | 27.809±1.69b | T(N)*** |
| | (Iso)leucine | 11.99±2.46a | 24.73±5.54b | 26.47±4.161b | 9.28±2.131c | 13.992±5.292 | 22.816±4.839 | 8.718±3.213 | 14.497±4.925 | T(N)* |
| | Leucine | 9.31±1.27ac | 34.52±12.01b | 28.09±6.33b | 8.31±1.46c | 11.935±4.497 | 15.415±4.575 | 8.278±3.455 | 12.835±4.541 | T(N)** |
| | Lysine | 5.05±1.75ab | 8.30±3.46ab | 10.24±0.94a | 3.26±0.83b | 10.52±2.17a | 11.146±0.20a | 4.291±0.96b | 3.006±0.97b | T(D)* |

| | | *A. thaliana* WT | | | | *PSP-17* mutant | | | | |
|---|---|---|---|---|---|---|---|---|---|---|
| | | **D** | **CdD** | **N** | **CdN** | **D** | **CdD** | **N** | **CdN** | **T-test: WT vs *PSP-17*** |
| | | | | | | | | | | |
| | Methionine | 2.35±0.169 | 2.09±0.318 | 1.86±0.141 | 1.89±0.114 | 2.615±0.298 | 2.736±0.71 | 2.741±1.101 | 3.965±1.987 | |
| | Phenylalanine | 16.41±2.851 | 12.12±0.941 | 23.56±2.46 | 19.56±4.636 | 20.318±4.133 | 14.968±2.946 | 17.086±2.478 | 14.75±5.346 | |
| | Serine | 440.42±82.41a | 410.04±93.21ab | 300.03±28.42a | 416.58±56.179b | 629.431±150.943a | 495.052±77.42a | 364.737±33.096b | 444.928±30.38a | |
| | Threonine | 140.50±16.70a | 163.35±15.64ab | 152.26±7.403a | 187.10±5.944b | 233.039±6.99a | 181.306±16.85bc | 180.351±5.81b | 181.643±10.19bc | T(D)*** |
| | Tryptophan | 2.28±0.287 | 1.56±0.3 | 1.63±0.499 | 1.99±0.465 | 1.629±0.29ac | 1.088±0.11b | 2.172±1.7ab | 6.109±5.08c | |
| | Tyrosine | 4.24±1.40a | 2.58±0.82a | 1.49±0.38b | 6.93±3.63c | 1.61±0.74a | 1.091±0.16a | 5.396±2.92b | 7.959±3.36b | T(D)*; T(N)* |
| | Valine | 39.30±5.340a | 76.92±15.74b | 63.60±11.823b | 36.84±5.80a | 44.418±7.187 | 61.672±13.921 | 33.739±6.795 | 34.65±12.506 | |
| **sugar acid** | Glyceric acid | 28.01±7.95a | 28.82±2.33ab | 8.28±0.29bc | 7.60±0.54c | 34.429±2.39a | 25.64±1.54b | 11.652±1.35c | 7.348±0.60d | T(N)* |
| **Sugar alcohols** | Glycerol | 360.32±59.64 | 336.16±78.73 | 429.19±41.68 | 389.67±23.388 | 403.328±34.07a | 374.246±49.9a | 367.574±43.65a | 277.736±17.11b | T(N)*; T(CdN)*** |
| | Mannitol | 8.58±1.61a | 8.26±0.86a | 4.49±0.77b | 4.51±0.57b | 7.327±1.30ac | 5.501±0.51ab | 4.71±0.16b | 7.131±0.56c | T(CdD)**; T(CdN)** |
| **Organic acids** | Gluconic acid | 1.43±0.528 | 0.94±0.231 | 1.38±0.243 | 1.59±0.396 | 1.097±0.104 | 1.384±0.449 | 1.868±0.618 | 1.724±1.051 | |
| | Hydroxyglutarate | 6.34±0.02a | 5.21±0.54a | 5.09±1.07a | 7.92±0.47b | 5.325±1.15a | 7.413±2.14a | 5.846±0.45a | 11.11±1.32b | T(CdN)* |
| | Maleic acid | 20.41±2.61a | 17.25±2.11a | 6.05±0.09b | 8.17±0.40a | 9.08±0.82ac | 7.158±0.43b | 10.034±0.70ac | 10.559±0.84c | T(CdN)* |
| | Malonic acid | 1.85±0.27a | 1.27±0.30b | 0.67±0.15a | 0.58±0.020a | 0.6±0.0415 | 0.677±0.0603 | 0.647±0.125 | 0.64±0.0946 | T(D)***; T(CdD)* |
| | Myoinositol | 419.40±58.15ab | 392.75±58.14a | 503.00±45.36ab | 514.76±49.52b | 586.904±171.02 | 437.89±12.29 | 555.903±59.63 | 514.424±85.03 | |
| | Putrescine | 22.79±4.514 | 31.34±6.781 | 28.29±6.935 | 30.33±3.309 | 24.429±9.258 | 18.082±1.252 | 28.315±3.682 | 17.831±0.782 | T(CdN)** |
